# Synergistic Cytotoxicity of Renieramycin M and Doxorubicin in MCF-7 Breast Cancer Cells

**DOI:** 10.3390/md17090536

**Published:** 2019-09-16

**Authors:** Jortan O. Tun, Lilibeth A. Salvador-Reyes, Michael C. Velarde, Naoki Saito, Khanit Suwanborirux, Gisela P. Concepcion

**Affiliations:** 1 The Marine Science Institute, University of the Philippines Diliman, Quezon City 1101, Philippines; lsreyes@msi.upd.edu.ph; 2 Philippine Genome Center, University of the Philippines Diliman, Quezon City 1101, Philippines; 3 Institute of Biology, University of the Philippines Diliman, Quezon City 1101, Philippines; mcvelarde@up.edu.ph; 4 Graduate School of Pharmaceutical Sciences, Meiji Pharmaceutical University, Tokyo 204-8588, Japan; naoki@my-pharm.ac.jp; 5 Department of Pharmacognosy and Pharmaceutical Botany, Faculty of Pharmaceutical Sciences, Center for Bioactive Natural Products from Marine Organisms and Endophytic Fungi (BNPME), Chulalongkorn University, Pathumwan, Bangkok 10330, Thailand; khanit.s@pharm.chula.ac.th

**Keywords:** renieramycin M, doxorubicin, synergistic combination chemotherapy, real-time profiling, cell cycle, gene expression profiling, apoptosis, breast cancer, blue sponge, DNA damage response

## Abstract

Renieramycin M (RM) is a KCN-stabilized tetrahydroisoquinoline purified from the blue sponge *Xestospongia* sp., with nanomolar IC_50_s against several cancer cell lines. Our goal is to evaluate its combination effects with doxorubicin (DOX) in estrogen receptor positive MCF-7 breast cancer cells. MCF-7 cells were treated simultaneously or sequentially with various combination ratios of RM and DOX for 72 h. Cell viability was determined using the MTT assay. Synergism or antagonism was determined using curve-shift analysis, combination index method and isobologram analysis. Synergism was observed with pharmacologically achievable concentrations of DOX when administered simultaneously, but not sequentially. The IC_95_ values of RM and DOX after combination were reduced by up to four-fold and eight-fold, respectively. To gain insights on the mechanism of synergy, real-time profiling, cell cycle analysis, apoptosis assays, and transcriptome analysis were conducted. The combination treatment displayed a similar profile with DNA-damaging agents and induced a greater and faster cell killing. The combination treatment also showed an increase in apoptosis. DOX induced S and G2/M arrest while RM did not induce significant changes in the cell cycle. DNA replication and repair genes were downregulated commonly by RM and DOX. p53 signaling and cell cycle checkpoints were regulated by DOX while ErbB/PI3K-Akt, integrin and focal adhesion signaling were regulated by RM upon combination. Genes involved in cytochrome C release and interferon gamma signaling were regulated specifically in the combination treatment. This study serves as a basis for in vivo studies and provides a rationale for using RM in combination with other anticancer drugs.

## 1. Introduction

The standard treatment for advanced breast cancer includes doxorubicin (DOX), administered either as monotherapy or in combination with other cytotoxic or targeted drugs [1]. However, its use has been limited because of dose-limiting toxicities such as cardiotoxicity. Moreover, the generation of drug-resistant tumors after continued therapy is still inevitable, and this eventually narrows down the treatment armamentarium. It is imperative therefore, to keep looking for new drug combinations that can enhance or maintain efficacy, while minimizing toxicity and delaying the development of drug resistance.

**Figure 1:**
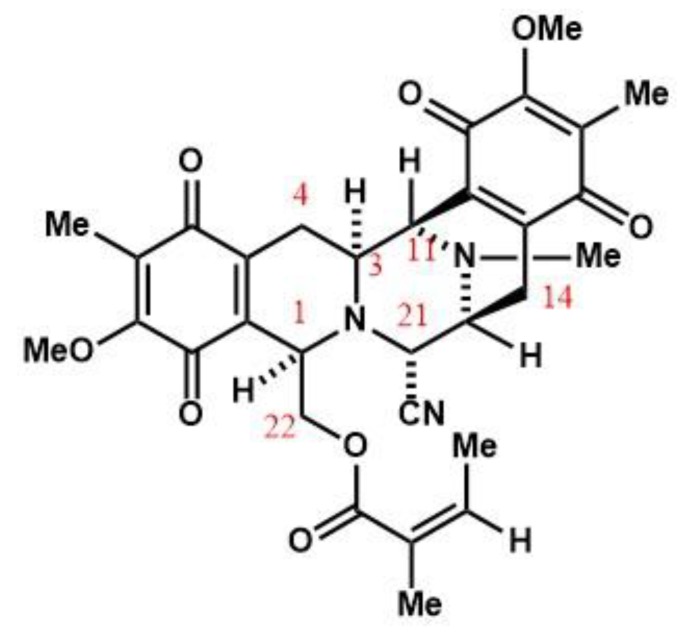
Renieramycin M from the blue sponge *Xestospongia* sp.

Renieramycin M (RM) is a KCN-stabilized tetrahydroisoquinoline purified from the blue sponge *Xestospongia* sp. (Figure 1), with nanomolar IC_50_s against the colon, lung, melanoma, and pancreatic cancer cells [2,3,4,5,6,7]. RM induces apoptosis and inhibits invasion and migration in non-small cell lung cancer cells (NSCLC) in vitro, making it a potential antimetastatic agent [8].

RM is structurally related to ecteinascidin-743 (Et-743; Trabectedin, Yondelis^®^), an anticancer drug for advanced soft tissue sarcoma and recurrent platinum-sensitive ovarian cancer. The renieramycins and ecteinascidins are the two major categories of the 1,2,3,4-tetrahydroisoquinoline alkaloids that have an anticancer effect. This warrants further investigation on the potential clinical utility of RM. A transcriptional structure–activity relationship (SAR) study and molecular network profiling revealed that RM and the ecteinascidin class of compounds induce apoptosis via a common pathway in the colon, breast [2], and glioblastoma cells [9]. Et-743 was reported to have a sequence-dependent synergistic effect with paclitaxel in breast carcinoma [10], and with doxorubicin in soft tissue sarcoma in vitro [11]. In view of the similarities between RM and Et-743, we hypothesize that RM can act also synergistically with standard cytotoxic drugs and thus, may be potentially useful to improve the therapeutic outcome.

In this study, we investigated the effects of the combination of RM and DOX in estrogen receptor positive (ER^+^) MCF-7, an in vitro model for the most common type of breast cancer and determined the drug ratio and regimen that will yield a synergistic effect. We also determined the effects of the combination on the cell cycle, apoptosis, and transcriptome in order to gain insights on the mechanism of combinatorial synergy, which could suggest therapeutic strategies for the treatment of breast cancer.

## 2. Results

### 2.1. RM Is More Potent Than DOX in MCF-7 Cells 

**Figure 2:**
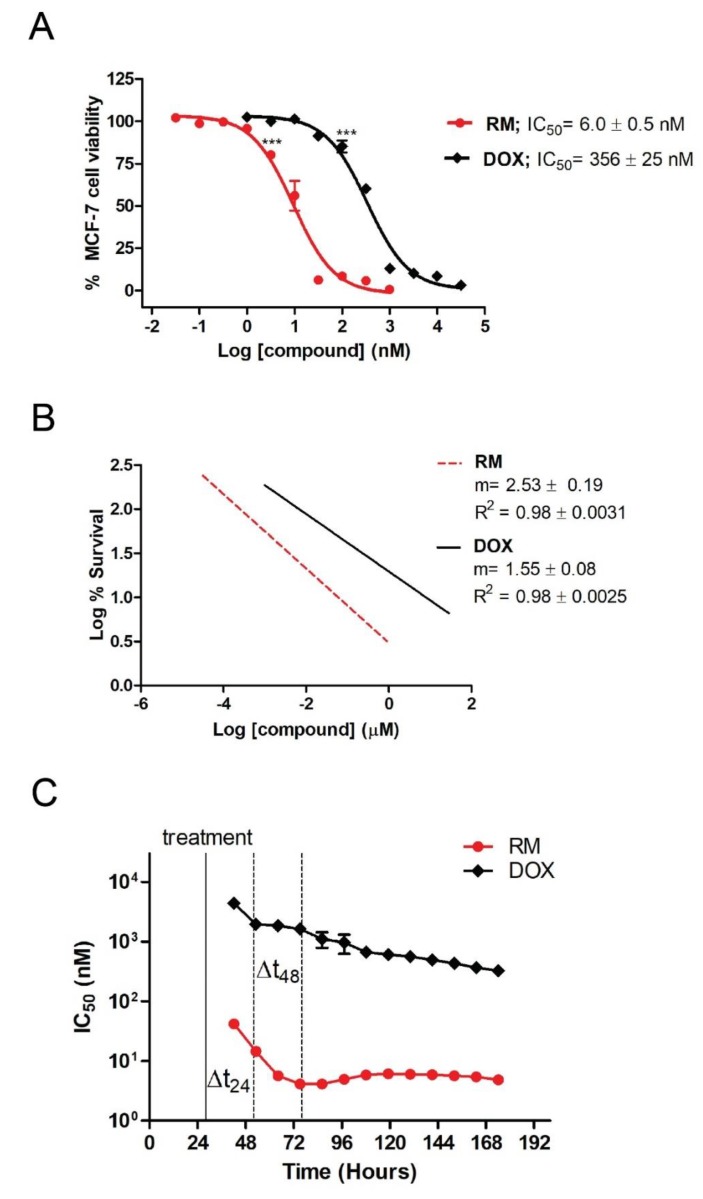
Individual cytotoxicity of renieramycin M (RM) and doxorubicin (DOX) on MCF-7 breast cancer cells. (**A**) Concentration-dependent cytotoxicity of RM and DOX from MTT cytotoxicity assay at 72 h post-treatment. Data points are mean ± SEM of three independent trials performed in quadruplicates. *** *p* < 0.0001 (one-way analysis of variance—ANOVA/Dunnett’s multiple comparison test). (**B**) Slopes of the concentration-response curves. m = “shape” or the slope of the curve; r = conformity of the data to the mass-action law. These were automatically generated using the CompuSyn software (Paramus, NJ, USA) [12]. (**C**) Time-dependent cytotoxicity of RM and DOX using xCELLigence^®^ software (RTCA; ACEA, Biosciences Inc., San Diego, CA, USA) during a seven-day exposure. Each data point represents mean IC_50_ ± SEM (*n* = 3) calculated during the indicated time-points of a representative experiment. Solid black line indicates the time of treatment; Δt_24_ and Δt_48_ indicate time points after 24 and 48 h of exposure, respectively.

The prerequisite for determination of synergistic activity is to know the potency and slope of the concentration-response curves of the individual drugs. Using MTT cytotoxicity assay, we determined the IC_50_ of RM and DOX in MCF-7 breast cancer cells after 72 h of exposure. Figure 2A shows the concentration-dependent cytotoxicity of the individual drugs, with RM being ~60-fold more potent (IC_50_ = 6.0 ± 0.5 nM) than DOX (IC_50_ = 356 ± 25 nM). Significant cytotoxicity was observed starting at 3.16 nM and 100 nM for RM and DOX, respectively. RM also shows a steeper sigmoidal curve compared to DOX as indicated by their slopes (m values; Figure 2B). Both compounds have R^2^ > 0.95 indicating an excellent linear correlation. 

We also monitored the effects of RM and DOX singly in real-time for a period of seven days and calculated the IC_50_ at different time points. The IC_50_ values of RM and DOX decreased over time indicating time-dependent cytotoxicity (Figure 2C, Figure S3). The IC_50_ of RM at all time points were lower than DOX, reflecting the more potent cytotoxicity of RM over DOX. RM also induces faster cytotoxicity (~24 h) than DOX (~48 h; Figure 2C). Interestingly, the cytostatic effect of DOX was observed after 24 h of exposure as shown by the brief lag in IC_50_. After 72 h, the IC_50_ of RM is almost constant, while that of DOX continued to gradually decrease.

### 2.2. RM Synergizes with DOX at Different Combination Ratios When Administered Simultaneously, But Not Sequentially in MCF-7 Cells

The next question that we addressed was whether RM could work synergistically with DOX. The combined effect of RM and DOX (RM + DOX) on MCF-7 breast cancer cells was investigated using an MTT cytotoxicity assay. First, cells were treated with RM and DOX at equipotent molar ratio (RM IC_50_/DOX IC_50_). Since RM was ~60-fold more potent than DOX (Figure 2A), a 1:60 constant ratio of RM:DOX was administered concurrently to the cells for 72 h. To demonstrate synergistic activity, multiple analytical approaches like the curve-shift analysis, combination index (CI), and isobologram analysis were performed. RM + DOX at the equipotent ratio (1:60) showed synergistic activity when ≥90% of MCF-7 cells were inhibited (Figure S4). 

The dosage and schedule of administration are important in combination chemotherapy since this can affect the therapeutic outcome. Depending on the ratio and schedule of administration (simultaneous or sequential), combination of cytotoxic compounds can yield a synergistic, additive, or antagonistic effect. This prompted us to determine the drug ratio and regimen that will yield maximum synergy. MCF-7 cells were treated simultaneously (RM + DOX) or sequentially with various constant molar ratios. For the sequential administration of compounds, cells were exposed either to RM first for 24 h, followed by DOX for 48 h (RM → DOX), or its reverse sequence, DOX for 48 h, followed by RM for 24 h (DOX → RM), without a washing step in between administrations (Figure S2). The exposure time was based on the real-time profile of the individual drugs, wherein RM required a shorter time to induce cytotoxicity than DOX (Figure 2C). To quantify synergistic effects, the CI values were calculated using the method of Chou and Talalay [13], where CI values < 1, =1, or >1 would mean synergistic, additive, or antagonistic activity, respectively. Table 1 shows the CI values at different inhibition levels (IC_50_–IC_95_) for each combination ratio and regimen tested.

In general, synergism was observed at high inhibition levels (≥90% inhibition), while additivity to antagonism was observed at lower inhibition levels (≤75% inhibition; Table 1). Synergism at high inhibition levels is more clinically relevant [14,15]. Among the three regimens, the highest synergistic effect was observed with the simultaneous treatment (Table 1). Sequential treatment of RM, followed by DOX (RM → DOX) yielded additive effect at high inhibition levels, while its reverse sequence (DOX → RM) showed an antagonistic effect (Table 1).

**Figure 3:**
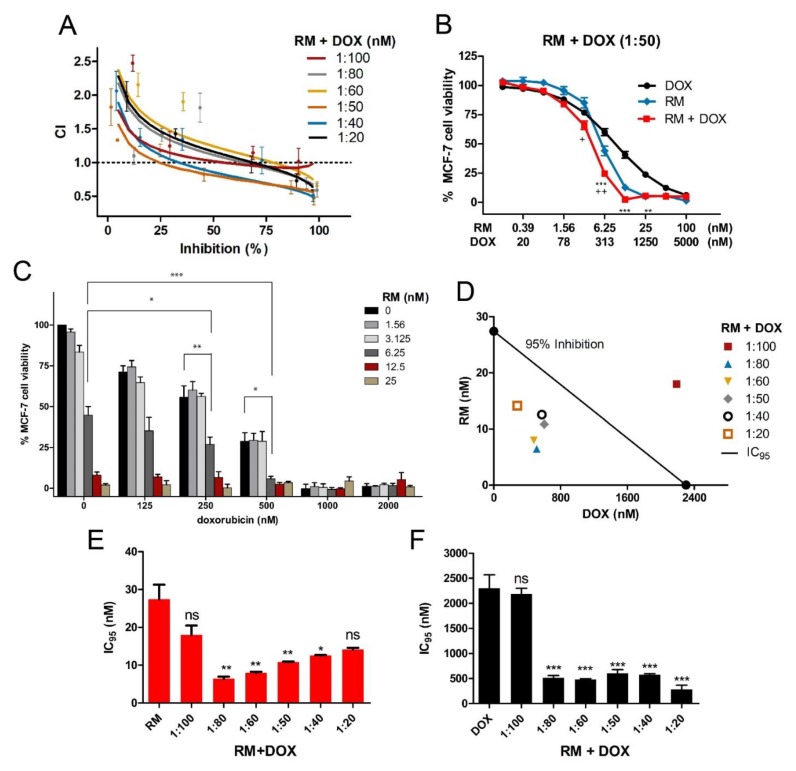
Synergistic cytotoxicity of RM + DOX at multiple combination ratios. MCF-7 cells were treated singly or concurrently with RM plus DOX at different constant molar ratios for 72 h, and the cell viability was assessed using an MTT cytotoxicity assay. (**A**) The combination index (CI) plot of different combination ratios. Values that fall below and above the line (CI = 1) would mean synergism and antagonism, respectively. Points are experimental data reported in mean ± SEM of three independent trials. (**B**) Example of curve-shift analysis conducted for the most synergistic combination ratio (1:50). Data points are mean ± SEM of three independent trials performed in quadruplicates * *p* < 0.05, ** *p* < 0.001, *** *p* < 0.0001, and + *p* < 0.05, ++ *p* < 0.001 vs. DOX alone and RM alone, respectively (one-way ANOVA/Bonferroni). (**C**) Comparison of cell viabilities in the checkerboard assay. Bars are mean + SEM of three independent trials. Significant enhancements in cytotoxicity were observed particularly when near IC_50_ of both drugs (RM 6.25 nM + 250 nM DOX (1:40) and 6.25 nM RM + 500 nM DOX (1:80)) were combined. * *p* < 0.05, ** *p* < 0.001, and *** *p* < 0.0001 (one-way ANOVA/Dunnett’s multiple comparison test). (**D**) Isobologram analysis of different combination ratios at IC95. The diagonal line denotes additivity at 95% inhibition. The isoboles are the mean of at least three independent trials. (**E**-**F**) Comparison of IC_95_ of single and combination drug treatments. The IC_95_ were computed using the CompuSyn software. The IC_95_ of the individual drugs from all sets of combination experiments were averaged and compared to the resulting IC_95_ after combination treatments. Each set of combination experiments was done at least three times. Data points reported are mean + SEM (*n* ≥ 3). RM (**E**) and DOX (**F**) IC_95_ were significantly lowered after using in combination at 1:80 to 1:20 ratio. * *p* < 0.05, ** *p* < 0.001, *** *p* < 0.0001, and ns—not significant (one-way ANOVA/Dunnett’s multiple comparison test).

**Figure 4:**
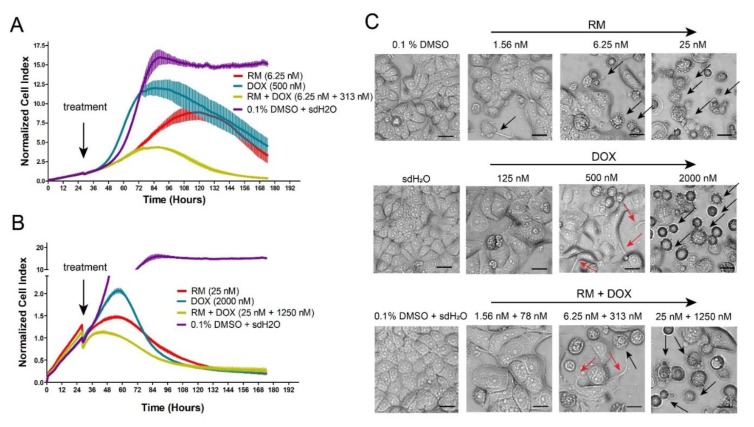
Dynamic monitoring of the combination effects of RM and DOX on MCF-7 breast cancer cells. Enlarged image of the kinetic cell response profiles of MCF-7 cells treated with combination of (**A**) 6.25 nM RM + 310 nM DOX or (**B**) 25 nM RM + 1250 nM DOX for seven days with the single drug equivalents juxtaposed for comparison. The cell indices were normalized at the time of compound addition indicated by the arrow. Data points are mean ± SD of (*n* = 4) of a representative trial. Full kinetic cell response profiles of MCF-7 cells treated singly and in combination (1:50) of RM and DOX for seven days are shown in Figure S6. (**C**) Morphological alterations in MCF-7 cells 72-h post treatment with RM + DOX (1:50). Magnification: 20×. Black arrows indicate apoptotic or dead cells. Red arrows indicate spikes peculiar to DOX-treated cells. Scale bar = 20 μm.

In the simultaneous treatment schedule, synergistic cytotoxicity was observed in multiple combination ratios, particularly within the 1:20 to 1:80 molar ratios, with the most synergistic effect observed at 1:50 and 1:40 (Figure 3 and Figure 4A). For the 1:50 and 1:40 combination ratios, synergistic effects were observed from IC_50_ to IC_95_ (Figure 3 and Figure 4A). In the CI plot, values that fell below and above the line (CI = 1) would mean synergism and antagonism, respectively (Figure 3A). The CI analysis is well supported by the curve-shift analysis showing significant enhancement of cytotoxic effects of both drugs as evidenced by the shift of the curve to the left relative to the single drug concentration-response curves (Figure 3B; Figure S5). The cytotoxicity of the individual drugs was significantly enhanced particularly when near IC_50_ or sub IC_50_ of each compound (3.13 to 6.25 nM for RM and 250 to 500 nM for DOX) were combined (Figure 3B,C, Figure S5). The synergistic activity is also reflected in the isobologram analysis (Figure 3D). The diagonal line in the isobologram is the line of additivity at 95% inhibition level. Similar to the CI plot, isoboles of the combinations that fall below and above the line of additivity indicated synergistic and antagonistic effects, respectively. In addition, combination of RM and DOX, lowered the concentrations required for both drugs to induce 95% inhibition (Figure 3D–F, Table S1). DOX IC_95_ was reduced by four- to 15-fold, while RM IC_95_ was reduced by two- to three-fold. (Figure 3D–F; Table S1). To investigate the mechanism of RM and DOX synergy, we used 1:50 ratio in the subsequent assays.

### 2.3. Combination of RM and DOX Displays Similar Profile with DNA-Damaging Agents with Accelerated and Increased Cytotoxicity

The effects of RM and DOX, singly and in combination, on MCF-7 cells were monitored in real-time using the xCELLigence^®^ System. This system utilizes a biosensor to assess compound-mediated cytotoxicity based on impedance [16]. The extent of change is proportional to the quantity, degree of attachment, and morphological alterations of the cells.

MCF-7 cells exposed to RM and DOX singly for seven days showed a concentration-dependent cytotoxicity indicated by the decrease in the cell index with increasing concentrations of RM and DOX (Figure S6A,B). This translated to sigmoidal concentration-response curves (Figure S6C), with IC_50_ values similar to those from the MTT cytotoxicity assay (Figure 2A). The cell index of vehicle treatments (0.1% DMSO for RM and sdH_2_O for DOX) increased over time indicating that the cells were viable (Figure S6A,B). Cells treated with 7.8 to 500 nM DOX showed a transient increase in the cell index higher than the vehicle treatment (Figure S6A; Figure 4A). This was followed by a decrease in the cell index over time. This kinetic pattern observed for DOX is a signature profile of DNA damaging agents such as topoisomerase inhibitors (e.g., etoposide, camptothecin, etc.) and anti-metabolites (e.g., 5-FU, and methotrexate) [17,18] that induce senescence [19,20,21]. In contrast, RM caused the same pattern, however, the transient increase in the cell index was lower than the vehicle treatment (Figure S6B; Figure 4A,B). This experiment was repeated at least three times, and similar results were obtained.

To validate this result, a parallel experiment on regular culture dishes were morphologically observed after 30, 48, and 72 h of exposure. RM and DOX (2000 nM) caused shrinkage, blebbing of membrane, and detachment of MCF-7 cells from the wells suggesting apoptotic cell death (Figure 4C; Figure S7A,B). In contrast, cells treated with near IC_50_ of DOX (125 to 500 nM) caused the cells to become enlarged and flattened with embossed nucleus and cytoplasmic protrusions (Figure 4C; Figure S7B), which are features of senescent cells [22,23]. These morphological alterations induced by DOX may be causing the initial increase in the cell index above vehicle treatments. The difference between the kinetic cell response profiles of RM and DOX therefore suggests that RM damages the DNA but does not induce senescence. This may also hint to a possible off-target or a different mode of action simultaneously taking place. The power of temporal resolution in predicting novel or off-targets of other compounds (i.e., monastrol and celecoxib) using a real-time cell analyzer (RTCA) has also been described [17].

Synergistic cytotoxicity was also evident in real-time. A greater decrease in the cell index values after combination treatment was observed, particularly with the following combinations: 6.25 nM RM + 313 nM DOX (Figure 4A) and 25 nM RM + 1250 nM DOX (Figure 4B). Moreover, a decrease in the cell index occurred at earlier time points indicating accelerated cytotoxicity when RM and DOX were used in combination. This was confirmed with morphological examination, with more dead cells observed in the combination than in the individual treatments (Figure 4C; Figure S7C).

The RM + DOX kinetic profile also resembled that of DNA-damaging agents (Figure 4A,B; Figure S6D); however, the transient increase in the cell index above control levels was not observed. This suggests an amplification of DNA damage, without recovery, and loss or reduction in the number of senescent cells. However, we do not exclude the possibility that other pathways may have contributed to the synergistic effect.

### 2.4. Combination of RM and DOX Increases Apoptosis

**Figure 5:**
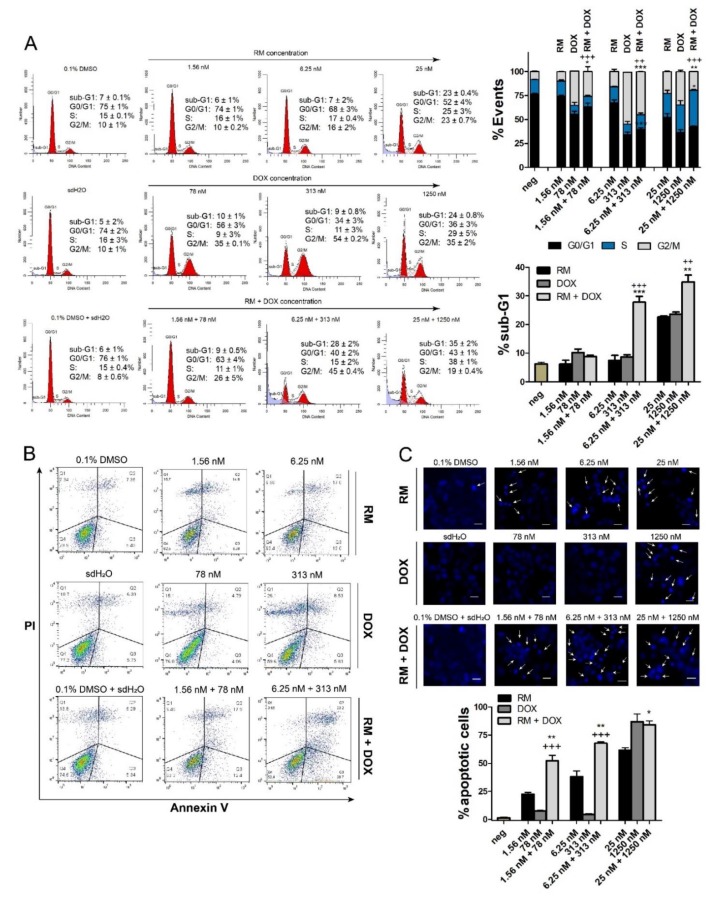
Combination effects of RM + DOX (1:50) on MCF-7 cell cycle and apoptosis. (**A**) DNA content analysis of MCF-7 treated singly or concurrently with RM and DOX for 72 h. The histograms are representative of two independent trials. The percentages reported are mean ± SEM and are represented also in bar graphs (right). High concentration of the combination (25 nM RM + 1250 nM DOX) induced an S and G2/M arrest, while lower concentrations of the combination (6.25 nM RM + 313 nM DOX and 1.56 nM RM + 78 nM DOX) only induced a G2/M arrest. Compared to the single drug treatments, a slight decrease in G2/M and a consequent increase in sub-G1 (dead cells) were observed after the combination treatment. MCF-7 treated with the vehicles 0.1% DMSO and sdH_2_O served as negative controls (neg). * *p* < 0.05, ** *p* < 0.001, *** *p* < 0.0001, and + *p* < 0.05, ++ *p* < 0.001, +++ *p* < 0.0001 vs. RM alone and DOX alone, respectively (one-way ANOVA/Bonferroni). (**B**) Annexin V/PI flow cytometry of MCF-7 treated singly or concurrently with RM and DOX for 30 h. The dot plots are representative figures of two independent trials. (**C**) Hoechst 33342 staining of nuclei after 24 h of exposure to the compounds. Shown are representative images of two independent trials. Minimum of 200 cells were counted manually and the number of apoptotic cells is reported in bar graphs (bottom). White arrows indicate brightly stained condensed nuclei. Bars are mean + SEM of three independent trials performed in quadruplicates. * *p* < 0.05, ** *p* < 0.001, and *** *p* < 0.0001. Scale bar = 20 μm.

Subsequently, we determined the effect of RM and DOX, singly and in combination, on the cell cycle. RM alone does not significantly affect the cell cycle. Only cells treated with high concentrations of RM (25 and 100 nM) induced late accumulation of cells in S and G2/M, which was only observed at 72 h (Figure 5A; Figure S8). In contrast, low concentrations of DOX alone (125 to 500 nM) showed a more pronounced G2/M arrest after 72 h without a significant increase in sub-G1 (Figure 5A, Figure S8). Meanwhile, high concentrations of DOX (2000 to 8000 nM) induced an arrest in S phase after 72 h, with a marked increase in sub-G1, suggesting that cells have undergone apoptosis. For the combination, higher concentration (25 nM RM + 1250 nM DOX) arrested the cells in S phase and slightly in G2/M, while lower concentrations (i.e., 6.25 nM RM + 313 nM DOX) arrested the cells in G2/M only (Figure 5A). These effects on the cell cycle may be attributed to DOX. Significant increase in sub-G1 was also observed (Figure 5A) and may have been induced both in a cell cycle-dependent and -independent manner.

Sub-G1 population is usually difficult to demonstrate in MCF-7, since this cell line is deficient in caspase 3, an enzyme responsible for DNA fragmentation [24,25]. For instance, the cell viability of MCF-7 at 72 h-post treatment with 25 nM and 100 nM RM was almost 5%–10% (Figure 2A), while sub-G1 was only at 23%–25% (Figure 5A). It is also possible that aside from apoptosis, another mode of cell death was induced by RM. To confirm apoptosis, we conducted Annexin V staining of the membrane and nuclear staining. Figure 5B,C shows an increase in the induction of apoptosis after simultaneous treatment of RM and DOX at 1:50 combination ratio compared to the individual drug treatments. This is evidenced by the increase in number of cells with condensed nuclei and Annexin V positive cells. The increase in apoptosis after the combination treatment was consistent with the real-time data (Figure 4) and MTT cytotoxicity assay (Figure 3).

### 2.5. Combination of RM and DOX Shows Signature Gene Expression Profile Unique from the Single Drug Treatments

To gain insights into the mechanism of synergism of RM + DOX, a microarray-based transcriptome analysis of MCF-7 treated with near IC_50_ of RM (6.25 nM), DOX (313 nM), and its combination (1:50) was conducted. We used the real-time kinetic profiles to determine a time point for comparative gene expression analysis. RNA was collected after 60 h, which is the onset of synergistic cytotoxicity (Figure 4B). At this time point, evident changes between treatments and the negative control were observed (Figure 4B).

**Figure 6:**
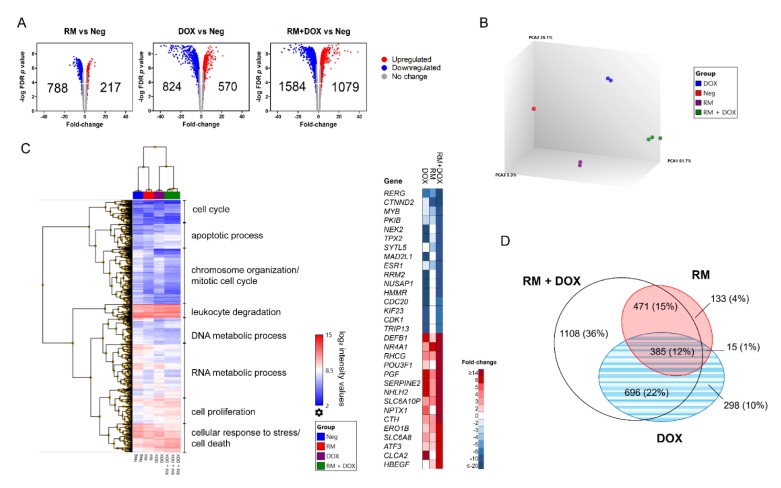
Transcriptome profile of MCF-7 treated with RM and DOX, singly and in combination. RNAs from MCF-7 cells treated with RM (6.25 nM) and DOX (313 nM) and its combination for 60 h were hybridized in an Affymetrix PrimeView Human Gene Expression Array. The single drug treatments and negative control (0.1% DMSO + sdH_2_O) were performed in duplicates while the combination treatment in triplicates. (**A**) Volcano plots showing the differentially expressed genes (DEGs). A threshold of two-fold upregulation (red) or downregulation (blue) relative to the negative control (Neg), with false discovery rate (FDR) *p* < 0.05 were used to filter the significant DEGs. (**B**) Principal component analysis and (**C**) hierarchical clustering of DEGs showing signature gene expression profiles of RM, DOX, and RM + DOX and the top 15 significantly enhanced or repressed genes upon combination treatment. (**D**) Venn diagram analysis showing the number of DEGs common or specific between treatments.

A total of 20084 genes were analyzed using the Affymetrix PrimeView Human Gene Expression Array. Genes with at least two-fold change in expression relative to the vehicle control (0.1% DMSO + sdH_2_O), with false discovery rate (FDR) *p* < 0.05 were considered differentially expressed genes (DEGs). The single drug treatments, RM and DOX induced 1005 (5%) and 1394 (7%) DEGs, respectively, while the combination treatment, RM + DOX induced the greatest number of DEGs, 2663 (13%), probably due to the synergistic effect (Figure 6A). More DEGs were downregulated both in single and combination treatments. A principal component analysis showed that RM, DOX, and RM + DOX had different signature gene expression profiles (Figure 6B). Hierarchical clustering indicated that the gene expression profile of the combination is more similar to DOX (Figure 6C) than RM. This may be due to the significant overlap of DEGs in RM + DOX combination with DOX (22%) than RM (15%; Figure 6D).

**Figure 7:**
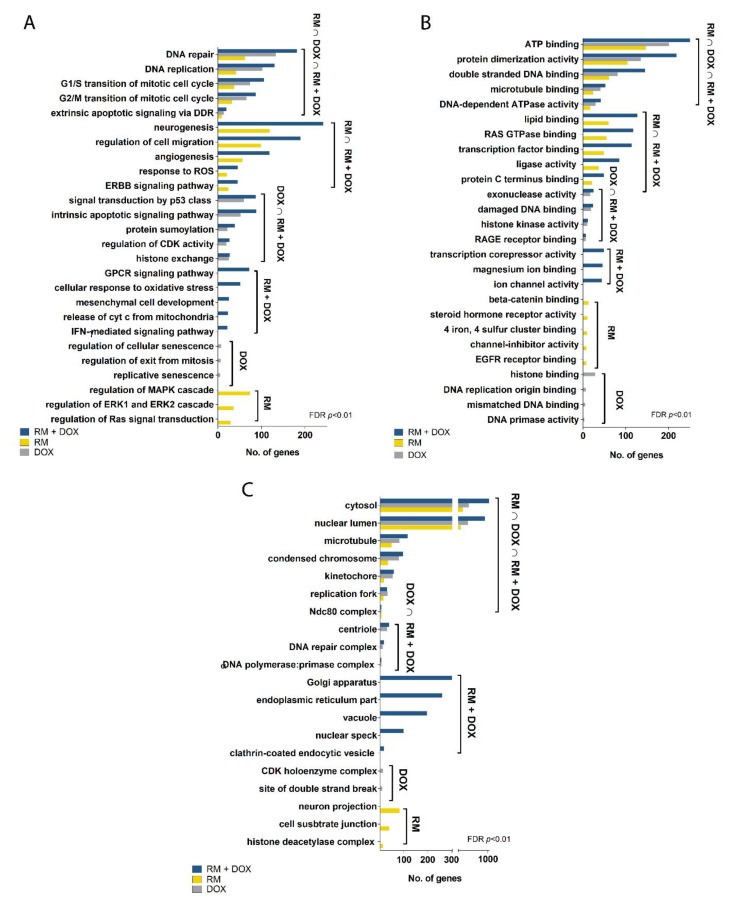
Gene ontology of the differentially expressed genes in MCF-7 treated with RM and DOX, singly and in combination. Statistical overrepresentation test with Benjamini–Hochberg correction was performed using GO, MSigDB, and QuickGO as reference databases. Only terms with FDR, *p* < 0.01 were considered significantly enriched. Shown are some of the most significant (**A**) biological processes, (**B**) molecular functions, and (**C**) cellular components as well as the number of genes associated with each term. The unabridged list of significant annotations is shown in Table S2.

We performed gene ontology (GO) statistical overrepresentation test (FDR, *p* < 0.01) to identify the biological processes, cellular components, and molecular functions associated with the DEGs. RM, DOX, and RM + DOX have common enriched annotations: DNA repair, DNA replication, cell cycle checkpoints in G1/S and G2/M, and extrinsic apoptosis signaling for biological processes (Figure 7A); ATP binding, protein dimerization activity, double-stranded DNA binding, microtubule binding, and DNA-dependent ATPase activity for molecular functions (Figure 7B); and cytosol, nuclear lumen, condensed chromosome, kinetochore, Ndc80 complex, and the replication fork for cellular components (Figure 7C). This suggests that RM and DOX have an overlapping mechanism of action that is activated both in the single and combination treatments. Taken together, the GO analysis reveals that both RM and DOX affect the DNA and stall the replication, then perturb the cell cycle, and finally induce cell death via apoptosis. The gene expression data corroborates with the real-time cytotoxicity data, showing the characteristic profile of DNA damaging agents (Figure 3), as well as with the cell cycle and apoptosis data (Figure 6).

Interestingly, there are gene ontologies that are common only between RM alone and RM + DOX (RM ∩ RM + DOX) and DOX alone and RM + DOX (DOX ∩ RM + DOX; Figure 7). For RM and RM + DOX, this includes neurogenesis, regulation of cell migration, angiogenesis, response to reactive oxygen species (ROS), and ErbB signaling pathway for biological processes; lipid binding, Ras GTPase binding, transcription factor binding, ligase activity, and protein C terminus binding for molecular functions. No unique cellular component was found for RM and RM + DOX. For DOX and RM + DOX, the common ontologies include signal transduction by p53 class mediator, intrinsic apoptotic signaling pathway, protein sumoylation, regulation of cyclin-dependent kinase (CDK) activity, and histone exchange for biological processes; exonuclease activity, damaged DNA binding, histone kinase activity, and RAGE receptor binding for molecular function; and centriole, DNA repair complex and α-DNA polymerase:primase complex for cellular components. The biological processes and molecular functions activated both in the single and combination treatments point toward the possible mechanism of synergistic cytotoxicity. Therefore, aside from activation of DNA damage response, the regulation of cell migration, angiogenesis, response to ROS, and ErbB signaling pathway activated by RM, and p53 signaling pathway and intrinsic apoptotic signaling activated by DOX, may be contributing to the observed synergistic effect. Other biological processes were uniquely enriched with the combination treatment and included G-protein coupled receptor (GPCR) signaling pathway, cellular response to oxidative stress, release of cytochrome c from mitochondria, and interferon gamma (IFN-γ) signaling pathway.

**Figure 8:**
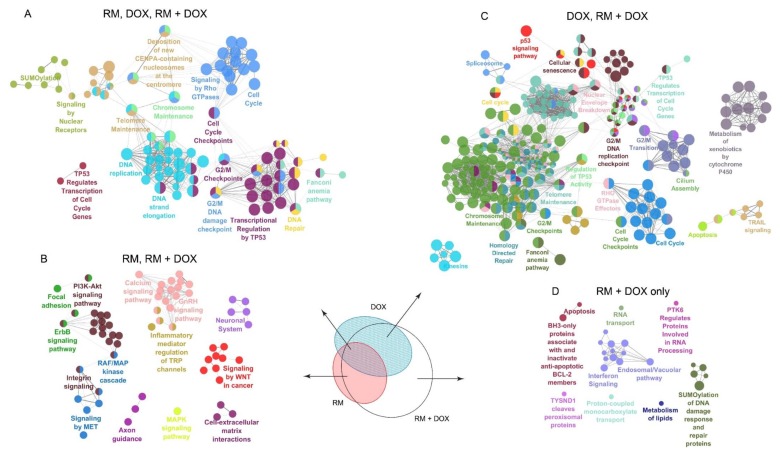
Network analysis of the pathways enriched in the synergistic cytotoxicity of RM and DOX. Kyoto Encyclopedia of Genes and Genomes (KEGG) and REACTOME pathways enriched by the DEGs that intersect between (**A**) RM ∩ DOX ∩ RM + DOX, (**B**) RM ∩ RM +DOX, (**C**) DOX ∩ RM + DOX, and those that are uniquely perturbed in (**D**) RM + DOX were determined using two-sided hypergeometric test with the Benjamini–Hochberg correction FDR, *p* < 0.05 and visualized as a network using ClueGO. Nodes represent pathways that are significantly enriched, the edges are kappa scores that measure the interrelatedness of the nodes. Unabridged list of the pathways can be found in Table S3.

To examine the mechanism of action further, we conducted a molecular networking analysis of the REACTOME and Kyoto Encyclopedia of Genes and Genomes (KEGG) pathways enriched by the DEGs common in single and combination drug treatment. We also looked at the pathways associated with the genes uniquely regulated in RM + DOX. The molecular networking analysis was consistent with the GO analysis (Figure 7). As expected, a large network involving mainly the DNA damage response and related cell cycle events were enriched in RM ∩ DOX ∩ RM + DOX. This suggests that RM and DOX collectively modulate the expression and activity of target proteins involved in DNA replication, DNA repair, G2/M cell cycle checkpoints, p53 regulation of transcription, telomere and chromosome maintenance, and sumoylation of proteins (Figure 8A).

**Figure 9:**
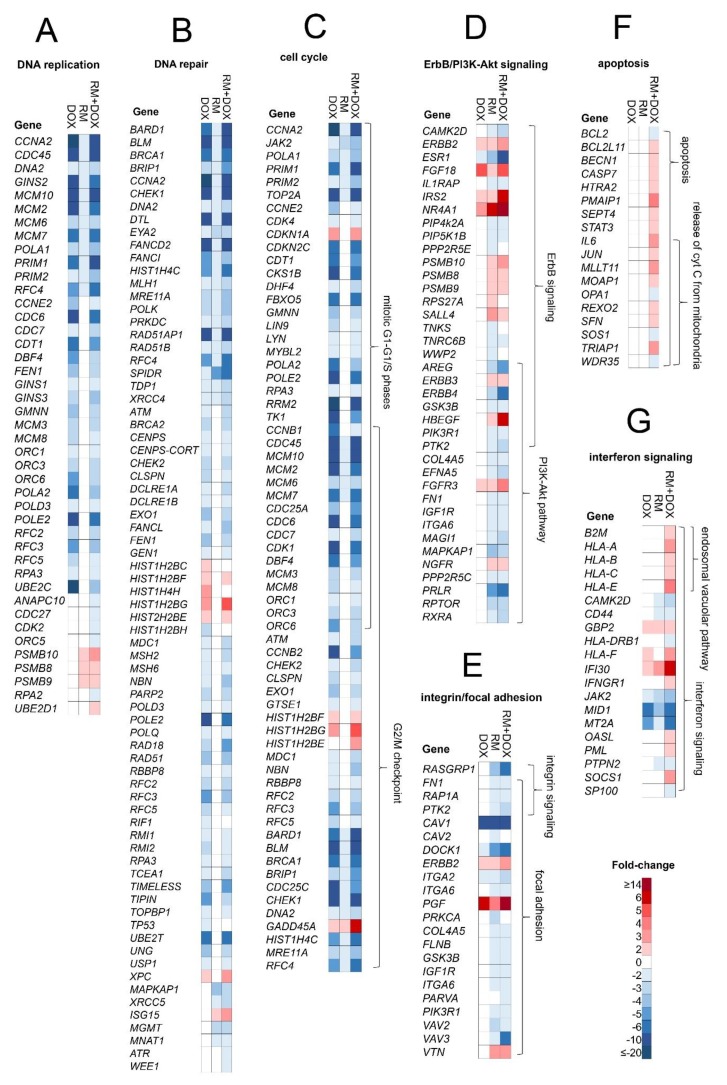
DEGs in MCF-7 after single and combination treatment with RM and DOX. Fold-change of genes involved in the following pathways are displayed as heat map: (**A**) DNA replication, (**B**) DNA repair, (**C**) cell cycle, (**D**) ErbB/PI3K-Akt signaling, (**E**) integrin/focal adhesion, (**F**) apoptosis, and (**G**) interferon signaling. The complete list of genes in all pathways enriched can be found in Table S3.

In RM ∩ RM + DOX, several pathways that are diverse from each other were enriched. One cluster includes the focal adhesion, PI3K-Akt pathway, ErbB pathway, RAF/MAPK cascade, integrin signaling, and signaling by MET, which are interrelated pathways that control cell survival. Another cluster involves secondary messenger signaling such as Ca^2+^ signaling pathway, gonadotropin-releasing hormone (GnRH) pathway, and inflammatory mediator regulation of transient receptor potential (TRP) channels. Signaling by Wnt and cell–extracellular matrix interactions were also activated (Figure 8B). In contrast, the genes uniquely induced or repressed in DOX ∩ RM + DOX were associated with a huge network that overlaps with the networks enriched in RM ∩ DOX ∩ RM + DOX, suggesting that DOX maybe reinforcing or amplifying the pathways affected by RM, and vice versa (Figure 8C). It is also important to note that cellular senescence is reflected in the gene expression profile of DOX, confirming the phenotypic response observed in real-time cell analyzer (Figure 4). Meanwhile, neurogenesis and axon guidance consistently appeared as a significant hit (FDR, *p* < 0.05) in the gene expression profile of RM (Figure 8A,C and Figure 9B), suggesting a possible neuroactivity. Interestingly, there are networks that are exclusively activated in RM + DOX only: Endosomal/vacuolar pathway, interferon signaling, apoptosis, and BH-3 only proteins that inactivate anti-apoptotic Bcl-2 proteins, RNA transport, and sumoylation of DNA damage response and repair proteins (Figure 8D).

The combination treatment was able to perturb simultaneously diverse pathways controlling cell survival: (1) DNA repair/replication, cell cycle, apoptosis by both RM and DOX, and (2) signaling by receptor tyrosine kinases (i.e., ErbB, PI3K-Akt), focal adhesion and integrin signaling by RM. We further investigated the genes involved in these pathways (Figure 9).

#### 2.5.1. DNA Replication

RM and DOX downregulated common genes involved in DNA replication: *CCNA2*, *CDC45*, *DNA2*, *GINS2*, *MCM2*/*6*/*7*/*10*, *POLA1*, *PRIM1/2*, and *RFC4* (Figure 9A) suggesting an overlap in the mechanism of action. These genes were significantly regulated both in single and combination treatments. DOX also downregulated several other genes: *CCNE2, CDC6/7, CDT1, GINS1/3, POLA2, POLD3, POLE2, RFC2/3/5,* etc. In contrast, RM upregulated the proteasome complex genes *PSMB8/9/10*. Interestingly, there are genes that were regulated exclusively after the combination treatment: *ANAPC10, CDC27, ORC5, RPA2, UBE2D1,* etc. Of particular significance is the replication protein A2 (RPA2), since this protein plays an important role in replication arrest. RPA2 defective cells conferred hypersensitivity to hydroxyurea treatment [26]. Taken together, these suggest that RM and DOX interfered with DNA replication via a different but related mechanism. Combination of the two drugs may have enhanced the DNA damage.

#### 2.5.2. DNA Repair

Both RM and DOX downregulated *BRCA1*, a key protein in double strand-break (DSB) repair via homologous recombination (HR) [27,28], and *ATM* and *CHEK1*, mediators and transducers of the DNA damage signal (Figure 9B). DOX also downregulated *BRCA2*, *CHEK2*, and poly(ADP-ribose) polymerase 1 and 2 (*PARP1/2*), resulting to additional DNA repair defects. Meanwhile, *ATR* was downregulated only in the combination, suggesting the occurrence of single strand breaks (SSB), possibly via DSB resection [29,30]. Downregulation of genes involved in ATM/ATR crosstalk (*MRE11*, *CTIP*, *EXO1*, *DNA2*) may have diminished ATR responses to DSBs [31,32,33,34]. Apart from *CHEK1/2*, *WEE1*, another G2 checkpoint kinase, was also downregulated in the combination, thus resulting to greater cell death.

#### 2.5.3. Cell Cycle

In response to DNA damage, cell cycle checkpoints are activated to prevent the progression of cells to the next phase allowing time for DNA repair. DOX regulated several p53 signaling proteins including TP53 (p53), GADD45A, and CDKN1A (p21; Figure 9B,C). p21 inhibited a series of cyclin-dependent kinases (CDKs), specifically CDK4 and CDK1 (Figure 9C), which blocked the cell cycle progression in the G1, S, and G2/M phases. This corroborates with the data on cell cycle analysis, RTCA, and the morphological examination (Figure 5 and Figure 6). In contrast, RM did not affect TP53 and CDKN1A, suggesting a p53-independent mode of apoptosis. This is contrary to the report of Halim et al. [8] and is probably due to the difference between the RM concentration (≥5 µM vs. 6.25 nM) used in the study. Moreover, RM was 10-fold more sensitive in p53 mutant MDA-MB-435 melanoma than in p53 wild type HCT116 colon carcinoma [2], supporting the hypothesis that p53 may not be required in the cytotoxicity of RM.

RM did not affect *CDK1/2/4/6*, which may explain the lack of pronounced effects in the cell cycle analysis. Among the CDKs and cyclins, only *CDK19*, *CCNA2*, *CCNB1IP1*, *CCNL1*, and *CCNY* were regulated by RM (Figure S9A). CDK19 is a regulator of triple-negative breast cancer (TNBC) showing a role in tumor initiation, proliferation, and metastases [35]. Inhibition of CDK19 by CDK19/8 inhibitor abrogated the mitogenic effect of estrogen on ER+ cell lines [36]. These data suggest the potential of RM in treating not only ER^+^ but also TNBC subtype as well. Consistent with a previous study [2], RM induced *GADD45A*, possibly via the reactive oxygen species (ROS) generated by its two quinone moieties [37]. Topoisomerase II α (*TOPIIA*), a direct target of DOX, was also downregulated by RM (2-fold), although not as much by DOX (19-fold), suggesting that this enzyme may not be a primary target of RM. 

The combination treatment did not affect *TP53* (Figure 9B), although the downstream effector kinases *CDKN1A* and *GADD45A* continued to be upregulated (Figure 9C), suggesting that some cells may have sustained the arrest induced by DOX. Interestingly, *CDK1*, the checkpoint for the M phase, became ~three-fold less downregulated after a combination treatment, suggesting that some cells may have progressed through mitosis despite the DNA damage.

#### 2.5.4. ErbB/PI3K-Akt Signaling

ErbB/PI3K-Akt pathway genes (e.g., *ERBB2/3/4*, *HBEGF*, *IGF1R*, *ESR1*, *PIK3R1*, *GSK3B*, and *PTK2*) were regulated by RM both in single and combination treatments (Figure 9D). These proteins are connected to the focal adhesion and integrin signaling (Figure 9E). *ERBB2/3* were upregulated, while *ERBB4* and its ligand, *HBEGF*, were downregulated and upregulated, respectively. *PIK3R1*, *ERBB2*, *ESR1*, *GSK3B*, and *PTK2* served as key hub molecules (Figure S9B). Tabunoki et al. [9] also reported the downregulation of ErbB (EGFR) and focal adhesion pathways by RM in U373MG glioblastoma cells. Transcription factors involved in estrogen mediated-signaling such as estrogen receptor 1 (*ESR1*) and V-myb myeloblastosis viral oncogene homolog (*MYB*) were among the top 15 genes with enhanced repression following combination treatment (Figure 6C).

#### 2.5.5. Apoptosis and Interferon Signaling

A group of apoptosis genes, several of them were specifically involved in the release of cytochrome C from mitochondria (e.g., *IL6, JUN, MLLT11, MOAP1, OPA1, SOS1*, and *TRIAP1*), were regulated exclusively after combination treatment (Figure 9F). Anti-apoptotic *BCL-2* was also repressed similar to the findings of Halim et al. [8]. This suggests that activation of intrinsic apoptosis via mitochondria could be related with the higher induction of cell death observed after simultaneous treatment of RM and DOX in MCF-7 cells. Interestingly, interferon signaling and endosomal vacuolar pathways were regulated in the combination treatment (Figure 9G), indicating immunomodulatory activity, which may have enhanced the DNA damage or cytotoxicity [38].

## 3. Discussion

The marine habitat proves to be a rich source of anticancer drugs [39]. Here we report another marine compound, renieramycin M (RM), from the blue sponge *Xestospongia* sp. that synergizes with DOX against ER^+^ MCF-7, thus offering potential for the treatment of the most common breast cancer subtype. This study serves as an initial attempt to assess the combination effects of RM with other standard cytotoxic agents, targeted, and immunotherapies.

The therapeutic outcome of drug combinations depends on the regimen, dosage or drug ratios, and mode of action of the compounds. For some class of compounds, certain ratios and schedule of administration are synergistic and others are antagonistic. Our results indicate that RM synergizes with pharmacologically achievable concentrations of DOX over a range of combination ratios (1:20–1:80, (RM:DOX)) when administered simultaneously and not sequentially. The synergistic effect was greatest at 1:40–1:50 drug ratio, particularly at 75%–95% kill level, which is most relevant in the clinics. Simultaneous administration of RM and DOX reduced the IC_95_ of both compounds by two- to eight-fold, hence toxicities are expected to be lower. 

We attempted to explain the mechanism of RM and DOX synergy by integrating real-time cell kinetic profiling, cell cycle, and transcriptome analysis. Data showed that RM and DOX damaged the DNA, possibly via different but overlapping mechanism of action. Both compounds repressed genes relating to DSB repair. DOX can (1) intercalate in the DNA minor groove, resulting in positive torsion, thereby inhibiting topoisomerase II (topo II) or (2) directly poison topo II in its double-strand cleavage form and prevent ligation [40,41]. RM also repressed topo II, but it was unlikely its main target. The basis of antiproliferative activity of RM is not well established, although mechanistic studies focusing on transcriptional profiling have shown similarities with the structurally related ecteinascidin compounds [2,9]. Et-743 binds to the exocyclic N2 amino group of guanines in the DNA minor groove via two of its rings (subunits A and B), forming DNA adducts and bending DNA toward the major groove [42]. Et-743 also interacts with transcription factors or DNA repair proteins via the third ring (subunit C) that protrudes from the DNA duplex [42,43]. In lieu of its chemical structure, it is also possible for RM to have a DNA and a non-DNA target, which would eventually trigger apoptosis. This is supported by the real-time cell profile and the transcriptome analysis revealing that RM downregulated DNA repair proteins and tyrosine kinase signaling proteins involved in ErbB/PI3K-Akt and focal adhesion pathways. The differential regulation of these pathways could be integral to the cytotoxicity of RM. Growth arrest and DNA damage-inducible gene 45 (*GADD45A*), a p53-inducible gene involved in the mitotic phase of the cell cycle, was induced by RM, corroborating the results of an earlier report [2]. However, our results revealed no significant cell cycle arrest, suggesting that other events have led to apoptosis. Possible routes could be through a putative protein target or the non-specific oxidative stress, which can cause DNA-strand breaks, membrane damage, and eventually, cell death [37]. Saframycin A (Saf A), another closely related tetrahydroisoquinoline with pyruvamide side chain at C-22 instead of an angelate ester [44], was also shown to alkylate guanine residues in DNA duplexes [45,46]. Both compounds offered compelling evidence supporting the involvement of iminium ion intermediate in the process, either by the expulsion of cyanide by Saf A or water by Et-743 [47]. However, based on gene expression analysis, Saf A did not affect DNA repair genes, as might have been expected if the primary action is through covalent modification of DNA [48]. Like Et-743, this suggests that Saf A also targets a non-DNA target, which was later on identified to be GAPDH via formation of DNA ternary complexes [49]. Based on the SAR study, the C-22 angelate ester is important in the cytotoxicity of RM [3] and may be key to the activation of DNA damage response. SAR studies designed to determine the effects on DNA, coupled with computational modeling will be important in unraveling the moieties that bind to the DNA and putative non-DNA targets. 

There are two possible mechanisms therefore to explain the synergy between RM and DOX in MCF-7 breast cancer cells. First, RM and DOX may have acted on the same or related pathway and amplified DNA injury while simultaneously repressing DNA repair pathways. One possible scenario would be accumulation of lesions due to DSBs and SSBs, requiring DNA repair. We hypothesized that these lesions were not repaired due to downregulation of BRCA1 by both compounds. With the simultaneous inhibition of PARP1/2 by DOX, the number of SSBs increased, resulting in a greater replication-associated DSBs, which in turn produced chromatid breaks and exchange aberrations, leading to cell death. Moreover, DOX also downregulated BRCA2, resulting to additional BRCA2-mediated HR repair defects. The mechanism of RM and DOX synergy may be reminiscent of the synthetic lethality in HR-defective breast and ovarian cancer (with BRCA1/2 mutations) treated with PARP inhibitors [27,28]. Another possible scenario would be the blocking of the mediators (ATM and ATR), and transducers (CHEK1 and CHEK2) of SSB and DSB repair. CHEK1/2 is responsible for controlling G1/S, S, and G2/M checkpoints. Simultaneous inhibition of CHEK1/2 and other DNA repair proteins may have propagated the DNA damage by permitting the replication of unrepaired DNA, causing genomic instability. Cells that accumulated DNA injury may have progressed through mitosis without arrest for repair and eventually underwent mitotic catastrophe or apoptosis [38]. Inactivation of anti-apoptotic BCL2 and cytochrome C release upon combination treatment may also have enhanced cell death. Finally, the activation of interferon gamma signaling may indicate immune regulation that may have altered the expression of DNA damage repair proteins and cell cycle regulators [38].

Second, RM and DOX may have acted on different targets or pathways that may either complement or reinforce drug action or neutralize compensatory mechanisms [50,51,52]. An example is vinblastine, a tubulin inhibitor that works synergistically with DOX for non-Hodgkin lymphoma [53] and triple-negative breast cancer [50]. Here we show that RM is able to downregulate ErbB (possibly through ERBB4), PI3K-Akt, and focal adhesion pathways. Miller et al. [54] reported that combined inhibition of CDK4 and IGF1R cooperatively suppresses the activation of proteins within the Akt pathway resulting in synergistic cytotoxicity. This might be implicated in the synergism of RM and DOX, as shown by the downregulation of IGF1R by RM, and CDK4 by DOX. Inhibition of PI3K-Akt by RM may also have utility in repressing DOX resistance in melanoma and colon cancer cells [55]. RM was reported to inhibit the migration and invasion of H460 lung cancer cells, thus may be a potential anti-metastatic agent [8]. We speculate that downregulation of focal adhesion and integrin signaling via protein tyrosine kinase 2 (PTK2) may be involved. PTK2 or focal adhesion kinase (FAK) is enriched in focal adhesions and together with Src kinase coordinates adhesion turnover, actin cytoskeleton dynamics and cell shape and regulates cancer cell migration and cell invasion [56]. Another key protein that was recently reported to be involved in breast cancer metastasis is the Myb [57]. A three- to five-fold increase in repression of MYB was observed after combination, thus providing another basis for its use in the treatment of advanced breast cancer. Currently, it is unclear whether perturbation of ErbB/PI3K-Akt and focal adhesion pathways occur upstream, downstream, or simultaneously with DNA damage response, and whether it may have impact to the synergistic effect of RM + DOX. Liu and Zhao [58] reported that two compounds with different gene expression profiles may offset each other’s effects when applied together, thus are less likely able to ‘collaborate’ to generate synergistic effects. Further investigation on the cross-talk of these two pathways is needed.

The mechanism of additivity and antagonism after sequential administration is currently unknown. We suspect that pretreatment with DOX have locked the cells at G2/M and caused senescence. Senescent cells tend to be resistant to apoptotic signals and may have reduced sensitivity to RM. Another possibility is that RM action may require dividing cells. A number of DEGs after RM treatment were associated with mitotic cell cycle, hence growth arrested cells may have reduced sensitivity to RM.

Several studies on the individual effects of RM and DOX have been published, however its combination effects have not been explored. To our knowledge, this is the first report on the synergistic cytotoxicity of RM and DOX in a breast cancer cell line with proposed mechanisms based on real-time kinetic profiles, cell cycle effects, and transcriptome signatures. Our study confirms several findings of previous reports and provides new insights to the mechanism of RM alone and the combination with DOX. However, the main drug driving the synergy remains largely unknown and further testing is needed. Although, it is possible that the mechanism of synergy may be explained by more than one mechanism. We found that the combination of RM and DOX also induces synergistic cytotoxicity in mammalian normal canine kidney cells (MDCK). This can be circumvented by synthesizing analogues of RM with improved selectivity. Other strategies such as antibody-drug conjugation [59] and controlled drug release in tumor tissues at specific ratios are being explored to maximize therapeutic index [60]. Successful translation of ratio-dependent synergistic drug combinations in pre-clinical and clinical trials using nano-scale drug delivery liposomes has been reported [61,62]. Currently, the efficacy of RM and DOX combination is being evaluated in vivo. Phenotypic profiling coupled with transcriptomic profiling of the compounds have aided us in understanding the synergism of RM and DOX. This study adds impetus to investigate the effects of RM with other anticancer drugs—cytotoxic agents, targeted, or immunotherapies, not only in ER^+^ but also in other subtypes of breast cancer and other solid tumors.

## 4. Materials and Methods 

### 4.1. Chemicals

The supply of renieramycin M (RM) for this study was isolated and purified using the method of Suwanborirux et al. [7]. RM is >99% pure. All NMR and MS data agree with the literature. A 10 mM (5.75 mg/mL) stock solution of RM was prepared in dimethyl sulfoxide (DMSO), and then further diluted to 100 µM using the same solvent. Doxorubicin (DOX), 98%–102% pure (HPLC), was purchased from Sigma-Aldrich Co. (Saint Louis, MO, USA). (D1515) and dissolved in sterile water to make 8.5 mM (5 mg/mL). Both stock solutions were aliquoted in several tubes, and stored at −20 °C. Further dilutions of RM and DOX were prepared in 10% DMSO and sdH_2_O, respectively. 

### 4.2. Cell Culture

MCF-7 (ATCC^®^ HTB-22™) was maintained following the supplier’s protocol. All cell culture products were purchased from Gibco^®^ (Waltham, MA, USA), unless otherwise stated. Briefly, cells were maintained at 37 °C under 5% CO_2_ in 10% MEM supplemented with 2 mM L-glutamine, 1× non-essential amino acids, 1 mM sodium pyruvate, 1.5 g/L sodium bicarbonate (Sigma-Aldrich^®^), 100 units of penicillin, and 100 μg/mL streptomycin, and 0.01 mg/mL recombinant human insulin (Sigma-Aldrich^®^). Culture media were renewed every two to three days, and cells were subcultured when 80%–90% confluent. Only cells that have >90% viability, passage number <20, and in the log phase of growth were used for assays. To harvest cells for assays, cells were washed three times with 1× PBS and incubated with 0.25% trypsin-EDTA at room temperature (RT; 24–26 °C) for 3–7 min or until the cells detached. Afterwards, cells were resuspended in 10% growth media. Seeding density was determined by manual counting in a hemocytometer using 0.4% trypan blue.

### 4.3. MTT Cytotoxicity Assay

Quantification of cell viability was based on an MTT (3-(4,5-dimethyl-2-thiazolyl)-2,5-diphenyl-2H-tetrazolium bromide) assay [63]. Briefly, cells were seeded into a flat bottom 96 well-plate at a density of 2 × 10^4^ cells/well and incubated at 37 °C under 5% CO_2_ for 24 h. Subsequently, cells were treated in quadruplicate with DOX (1 to 31600 nM), RM (0.0316 to 1000 nM) or vehicle controls (0.1% DMSO and sdH_2_O) and exposed for 72 h. Then, 15 μL of filtered MTT (5 mg/mL in 1× PBS) was added to each well and incubated for another 3 h. Finally, 100 μL of DMSO was added, and the absorbance was read in a microplate reader (Biotek^®^, Synergy-HT^®^, Winooski, VT, USA) at 570 nm. The percent cell viability relative to the vehicle control was calculated using the following equation:

$$\%\;Cell\;Viability\;=\left(\frac{Absorbance\;compound\;treated\;–\;Absorbance\;media}{Absorbance\;vehicle\;treated\;–Absorbance\;media}\right)100.$$

The IC_50_ and slope (m) of the concentration-response curves were calculated using the CompuSyn software developed by Chou and Martin [12]. These values were then used for the evaluation of the drug combination experiments described below.

### 4.4. Real-Time Monitoring of Cytotoxicity 

This assay utilized the xCELLigence^®^ real-time cells analyzer to monitor the effects of the compounds to the cells. Cell titration was first conducted to determine the appropriate seeding density in the assay (Figure S1). To start the real-time cell analysis, background readings from 100 μL of media added in each well of the E-plate^®^ 96 was obtained. A 100 μL aliquot of the cell suspension (2 × 10^4^ cells/well) was added, and the plate was equilibrated at RT for 30 min before placing in the RTCA-SP station inside the 5% CO_2_ incubator. After ~24 h, the cells were treated with the compounds in the same manner as in the MTT cytotoxicity assay. Cellular responses were monitored every 30 s for 1 h, then every 2 min for another 1 h, and finally every 30 min for 144–168 h (six to seven days). Cell index values were normalized to the time of addition of compounds and plotted in GraphPad PrismTM version 3.03 (San Diego, CA, USA). IC_50_s were calculated using the sigmoidal-dose response curve-variable slope equation using the RTCA-SP software version 2.0 (San Diego, CA, USA).

### 4.5. Cell Cycle Analysis

This assay was based on Riccardi and Nicolletti [64] with slight modification. Briefly, MCF-7 cells were grown in six-well plates at 6.7 × 10^5^ cells/well. After 24 h, cells were treated, either singly or in combination, with DOX (125 to 8000 nM) and RM (1.56 to 100 nM), then harvested at 30 and 72 h-post incubation to generate a concentration- and time-dependent response data. Cells were washed with 1 mL cold 1× PBS and centrifuged at 200g (Thermo Scientific, Heraeus™ Megafuge 16R, Waltham, MA, USA) for 5 min at RT. Cells were resuspended in 500 μL of cold 1× PBS then fixed for a minimum of 24 h by adding 4.5 mL of 70% cold ethanol. Afterwards, the cell suspension was spun at 800 g for 10 min to remove the ethanol. Cells were washed with 5 mL 1× PBS, spun at 800 g for 10 min, and resuspended in 500 μL of 1× PBS. Subsequently, 500 μL of 0.192M Na_2_HPO_4_, pH 7.8, with 0.004% Triton X-100 was added, incubated at RT for 5 min, and centrifuged at 800g for 10 min. Cells were resuspended in 1 mL of freshly prepared DNA staining solution (200 μg of propidium iodide (PI) in 10 mL 1× PBS, with 2 mg of DNAse free RNAse (Roche^®^, Basel, Switzerland) and incubated at RT in the dark for 4 h. Finally, cells were analyzed using flow cytometry (FACS Calibur, BD Biosciences, Tuas, Singapore) at fluorescence channel 2 (FL-2; λ_ex_ 488 nm, λ_em_ band pass filter 550 nm). Cell cycle analysis was performed using ModFt LT 5.0 software (Verity Software House, Topsham, ME, USA).

### 4.6. Evaluation of Sequential or Simultaneous Administration of RM and DOX 

For the drug combination studies, the diagonal constant ratio scheme by Chou [13] was used. The effect of the drug combination was evaluated first at the equipotent molar ratio, RM:DOX (1:60), which is based on the IC_50_ values of the individual drugs obtained from the MTT cytotoxicity assay. MCF-7 cells were treated simultaneously with RM (0.2 to 100 nM) plus DOX (12 to 6000 nM) in quadruplicates at a constant 1:60 ratio for 72 h. The effect on cell viability was evaluated by MTT cytotoxicity assay. DMSO (0.1%) + sdH_2_O served as vehicle control. To monitor the behavior of the individual drugs during the combination experiment, a parallel single drug control experiment for RM and DOX was performed. We used three analytical approaches to identify synergistic activity: Curve-shift analysis, combination index (CI) method, and isobologram analysis. The CI values at different inhibition levels and the IC_50_, IC_75_, IC_90_, and IC_95_ used to construct the lines of additivity were generated using the CompuSyn software [12]. 

Subsequently, we determined the optimal combination ratio and regimen that will give maximal synergistic effect on MCF-7. Cells were exposed to multiple combination ratios (e.g., 1:100, 1:80, etc.) at three different schedules (Figure S2): (1) Simultaneous treatment (RM + DOX), (2) sequential treatment (RM→DOX after 24 h), (3) reverse sequence (DOX→RM after 48 h). The total time of exposure for each regimen was 72 h. The exposure times of each drug in sequential treatments were based on the cell killing kinetics of the individual drugs from xCELLigence RTCA. For the determination of optimum combination ratio, a checkerboard assay was also performed to complement the diagonal constant ratio method. All experiments were performed at least three times.

### 4.7. Hoechst 33342 Nuclei Staining

MCF-7 cells were grown in flat-bottom 96-well plates at a density of 2 × 10^4^ cells/well. After 24 h, cells were treated in triplicates with RM alone, DOX alone, or in combination at 1:50 molar ratio (most synergistic combination ratio) for 24 h at 37 °C. Vehicle solvent (0.1% DMSO + sdH_2_O) served as negative control. Afterwards, cells were washed with 1× PBS once, and the media were replaced. Cells were stained in situ with Hoechst 33342 (5 μg/mL in sterile H_2_O; Invitrogen) for 20 to 30 min at 37 °C. Finally, cells were excited with UV, and viewed under a fluorescence microscope at λ_em_ ~483 nm. At least 200 cells were counted manually, and the percent apoptotic cells were reported.

$$\%\;apoptotic\;cells\;=\left(\frac{no.\;of\;apoptotic\;cells}{total\;no\;of\;cells}\right)100.$$

### 4.8. Annexin V/PI Flow Cytometry

This assay utilized Annexin V-FITC Apoptosis Detection Kit (BD Pharmingen™, Tuas, Singapore) and was done according to the manufacturer’s protocol, with slight modification. Cells were grown in six-well plates at 6.7 × 10^5^ cells/well. After 24 h, cells were treated with RM alone, DOX alone, or in combination at 1:50 molar ratio for 24 h at 37 °C. Vehicle solvent (0.1% DMSO + sdH_2_O) served as negative control. Subsequently, cells were washed once with cold 1× PBS, spun at 300 g and resuspended in 600 μL of 1× binding buffer yielding ~1 × 10^6^ cells/mL. Then, 100 μL of the cell suspensions were stained with 5 μL Annexin-V and 5 μL propidium iodide (PI). After 15 min of incubation at RT in the dark, 400 μL of 1× binding buffer (140 mM NaCl, 4 mM KCl, 0.75 mM MgCl_2_, 10 mM HEPES, and 1.75 mM CaCl_2_) [65] were added to each tube. Finally, cells were analyzed using FACS Calibur at FL-1 (λ_em_ ~483 nm) and FL-2 (λ_em_ ~483 nm) channels. The experiment was performed in two independent trials. A total of 10000 cells were interrogated and the percentages per quadrant were computed using FlowJo v10 software (Ashland, OR, USA).

### 4.9. Microarray-Based Transcriptome Analysis

MCF-7 cells were treated with near IC_50_ concentrations of RM (6.25 nM), DOX (313 nM), and its combination for 60 h. Individual drug treatments and vehicle control were prepared in duplicates, while the combination treatment was prepared in triplicates. Cells were lysed using QIAshredder (Qiagen, Hilder, Germany), followed by RNA extraction using QIAgen RNeasy mini kit (Qiagen, Hilder, Germany) and sent to Origen Labs Inc. (Ayer Rajah Crescent, Singapore) within 24 h for microarray hybridization using Affymetrix PrimeView Human Expression Arrays. CEL files were generated using Affymetrix GCC (GeneChip Command Console) 5.0 software (Waltham, MA, USA) and imported to the Transcriptome Analysis Console (TAC 4.0) software (Waltham, MA, USA) for data quality control (QC). RMA normalization were performed on the samples to generate the QC metrics. Samples showed OD_260_/OD_280_ ratios of ~1.793 to 1.915, with concentrations of 132.81 ng/μL to 541.99 ng/μL, indicating good purity and acceptable concentrations.

Mean absolute relative log expression (RLE), principal component analysis (PCA) plot and hierarchical clustering of differentially expressed genes (DEG) were conducted in TAC 4.0. CHP files were used to obtain DEGs using a one-way analysis of variance—ANOVA (eBayes) and filter criteria of fold change ≤−2 or ≥2 with an ANOVA, *p* < 0.05. Gene ontology (GO) of the DEGs was conducted using ClueGO v. 2.5.1 (Paris, France) [66]. The Molecular Signature Database (MSigDB) C5 Collection v. 6.2 of the Broad Institute (updated July 2018) (Cambridge, MA, USA) [67] and QuickGO of EMBL-EBI (updated August 2018) (Hinxton, Cambridgeshire, UK) [68] were used as reference gene lists. Enrichment/depletion (two-sided hypergeometric test) with Benjamini–Hochberg correction was performed. Only hits with false discovery rate (FDR) *p* < 0.01 were considered statistically significant. Annotation data sets used were GO complete biological process, cellular component, and molecular function. ClueGO was also used for pathway enrichment and network analysis with REACTOME pathways (updated August 2018) [69] and Kyoto Encyclopedia of Genes and Genomes (KEGG) pathways (updated August 2018) [70] as reference lists. Other databases that use different algorithm from ClueGO, such as Gene Ontology Consortium v. 13.1 (released 2018-02-03) [71,72] and Protein Annotation Through Evolutionary Relationship (PANTHER; updated August 2018) [73] were also utilized to validate the results. An overrepresentation test using Fisher’s exact test with FDR correction was performed. Terms with FDR, *p* < 0.05 were considered significant. Venn Diagrams were constructed using EulerAPE (University of Kent, Canterbury, UK) [74]. Microarray gene expression data (GSE124597) were submitted to Gene Expression Omnibus (GEO).

### 4.10. Statistical Analysis

All experiments were performed with at least two to three independent trials. Results were expressed as mean ± SD or mean ± SEM. Statistical comparisons were performed using GraphPad Prism^TM^ version 3.03 using one-way ANOVA (analysis of variance) followed by Bonferroni or Dunnett’s multiple comparison test or unpaired two-tailed Student’s *t*-test. *p* < 0.05 were considered significant.

## 5. Patents

This study has been the subject of patent application by J.O.T. and G.P.C. of the Marine Science Institute, University of the Philippines Diliman with patent application number 1-2018-050034, patent pending.

## Figures and Tables

**Table 1:** Heat map of the effects of RM and DOX at different combination ratios and regimen.

Drug Combination	Combination Ratio (nM)	n	CI values at			
			IC_50_	IC_75_	IC_90_	IC_95_
RM + DOX	1:100	5	0.956 ± 0.064	0.886 ± 0.047	0.884 ± 0.111	0.917 ± 0.156
RM + DOX	1:80	3	1.111 ± 0.093	0.913 ± 0.047	0.777 ± 0.056	0.712 ± 0.085
RM + DOX	1:60	3	1.202 ± 0.115	1.011 ± 0.075	0.877 ± 0.054	0.810 ± 0.065
RM + DOX	1:50	5	0.806 ± 0.069	0.685 ± 0.044	0.609 ± 0.036	0.575 ± 0.041
RM + DOX	1:40	3	0.856 ± 0.041	0.691 ± 0.048	0.580 ± 0.061	0.524 ± 0.071
RM + DOX	1:20	3	1.160 ± 0.075	0.942 ± 0.047	0.780 ± 0.033	0.692 ± 0.032
RM → DOX	1:100	6	2.372 ± 0.220	1.601 ± 0.156	1.105 ± 0.121	0.870 ± 0.105
RM → DOX	1:20	4	1.414 ± 0.094	1.173 ± 0.087	1.004 ± 0.132	0.918 ± 0.162
DOX → RM	100:1	5	1.622 ± 0.221	1.545 ± 0.209	1.557 ± 0.311	1.620 ± 0.427
DOX → RM	20:1	3	2.543 ± 0.429	2.273 ± 0.288	2.074 ± 0.263	1.974 ± 0.319

The effects on cell viability were determined using an MTT cytotoxicity assay after 72 h, and the combination index (CI) values were calculated using CompuSyn software. To determine the combination ratio and schedule of administration that yield the greatest synergistic effect, a heat map was generated, with the red color highlighting the lowest CI values signifying synergism, white highlighting values near 1 signifying additivity, and blue highlighting values greater than 1 signifying antagonism. RM + DOX indicates simultaneous administration, RM → DOX indicates pre-exposure to RM for 24 h followed by DOX for 48 h and DOX → RM indicates the reverse order. *n* is the number of independent trials. Each trial consisted of single drug controls and their combination. The CI values are mean ± SEM of n trials.

## Supplementary Materials

The following are available online at https://www.mdpi.com/1660-3397/17/9/536/s1, Figure S1: Real-time monitoring of the density-dependent proliferation of MCF-7 cells for 6 days, Figure S2: Treatment protocol for the evaluation of synergistic activity, Figure S3: Time-dependent cytotoxicity of RM and DOX using xCELLigence real-time cell analyzer (RTCA), Figure S4: Synergistic cytotoxicity of RM + DOX at equipotent molar ratio (1:60), Figure S5: Curve shift-analyses of the different combination ratios of RM + DOX, Figure S6: Dynamic monitoring of the effects of RM and DOX, singly on MCF-7 breast cancer cells, Figure S7: Morphological changes in MCF-7 breast cancer cells treated singly, and in combination of RM and DOX for 30 and 48 hrs, Figure S8: Effects of RM alone and DOX alone on MCF-7 cell cycle, Figure S9: CDKs and cyclins and molecular networking, Table S1: Comparison of IC_50_ and IC_95_ of single and combination drug treatments.
