# Supplementary material for: Synergistic Cytotoxicity of Renieramycin M and Doxorubicin in MCF-7 Breast Cancer Cells
Source: Mar Drugs. 2019 Sep 16;17(9):536. doi: 10.3390/md17090536 (PMC6780817; doi:10.3390/md17090536)
Supplement: Supplementary file 1 [file marinedrugs-17-00536-s001.zip › Supplementary Information.docx]

**Supplementary Information**

**
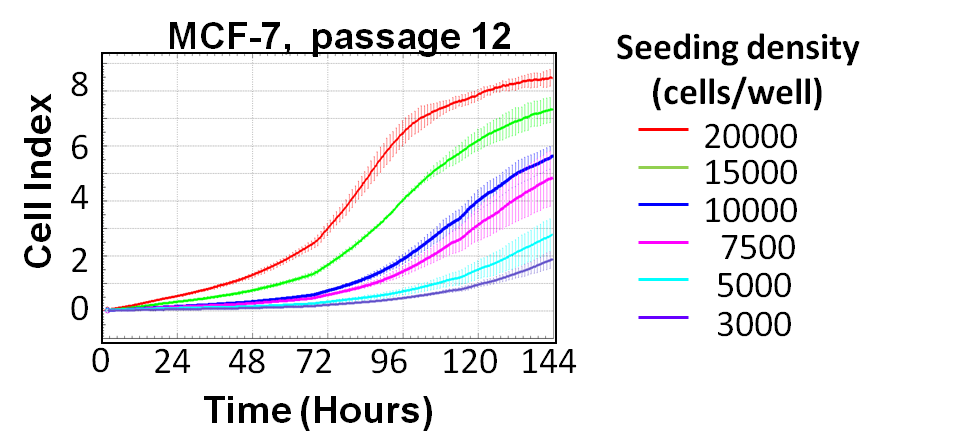
**

**
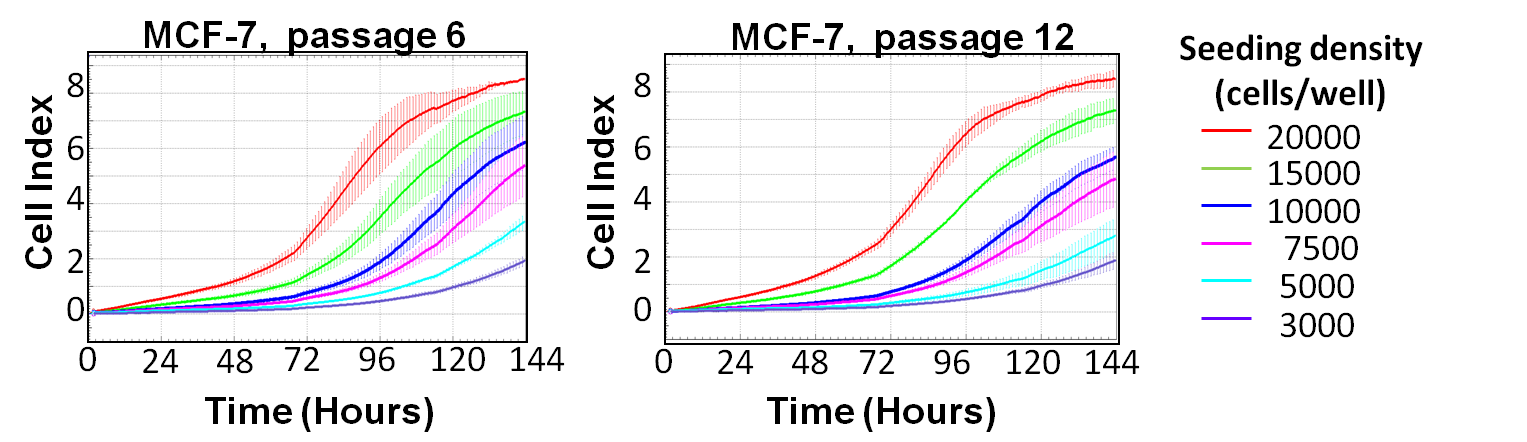
**

**Figure S1. Real-time monitoring of the density-dependent proliferation of MCF-7 cells for 6 days.** MCF-7 cells (passage no 12 and 6) were titrated to determine the correct seeding density that will be used in the xCELLigence system. The kinetic profiles of MCF-7 cells were consistent in different passage numbers.

**
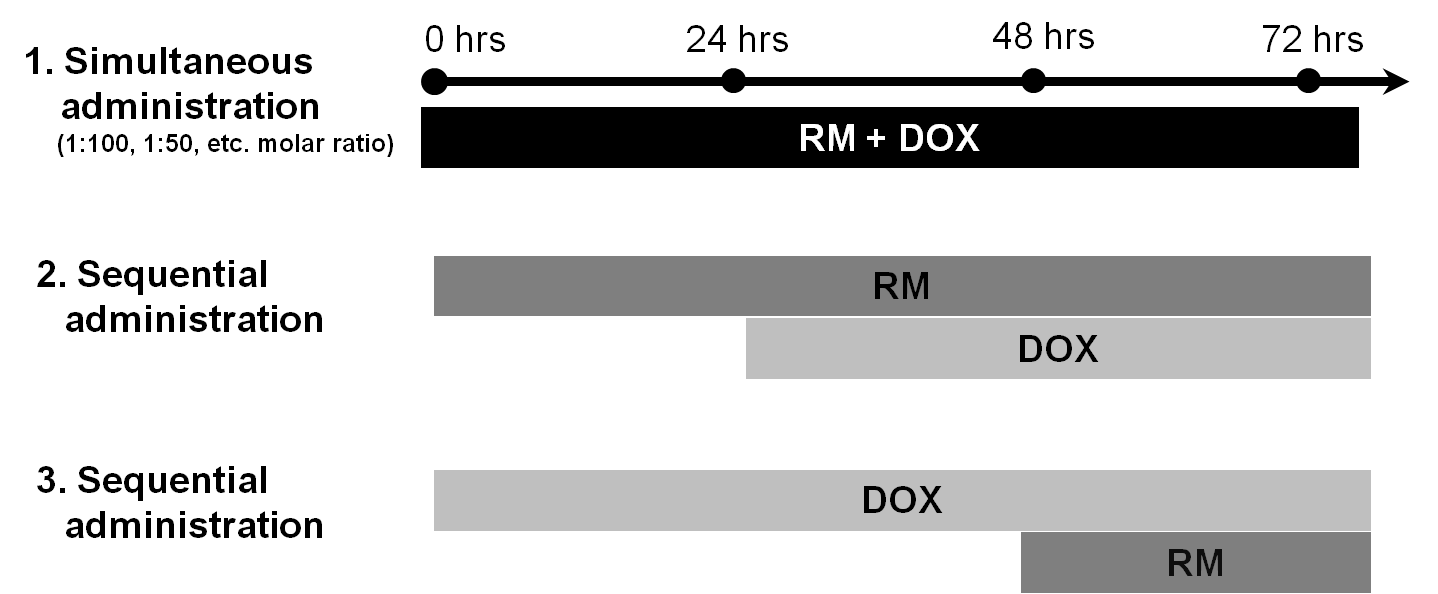
**

**Figure S2. Treatment protocol for the evaluation of synergistic activity.**

**
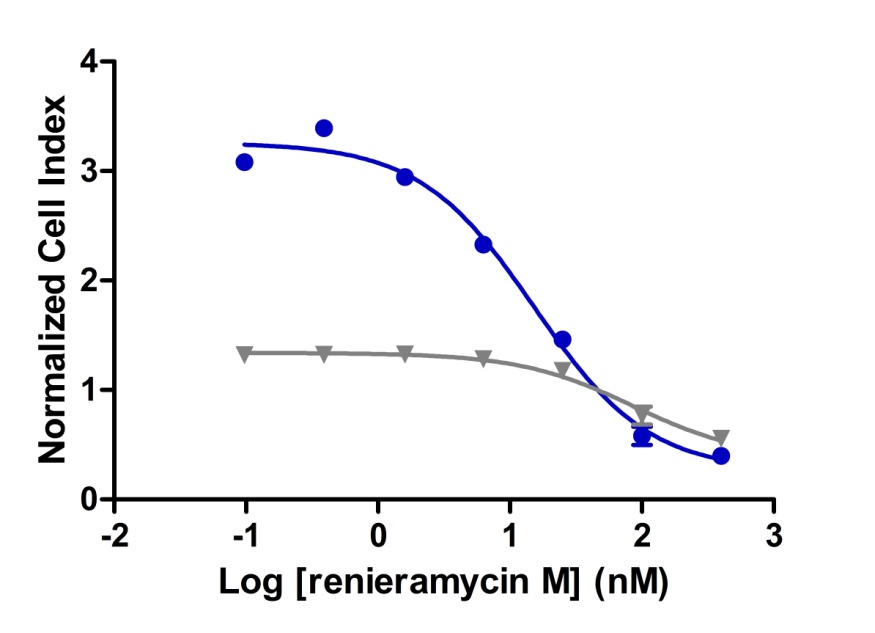

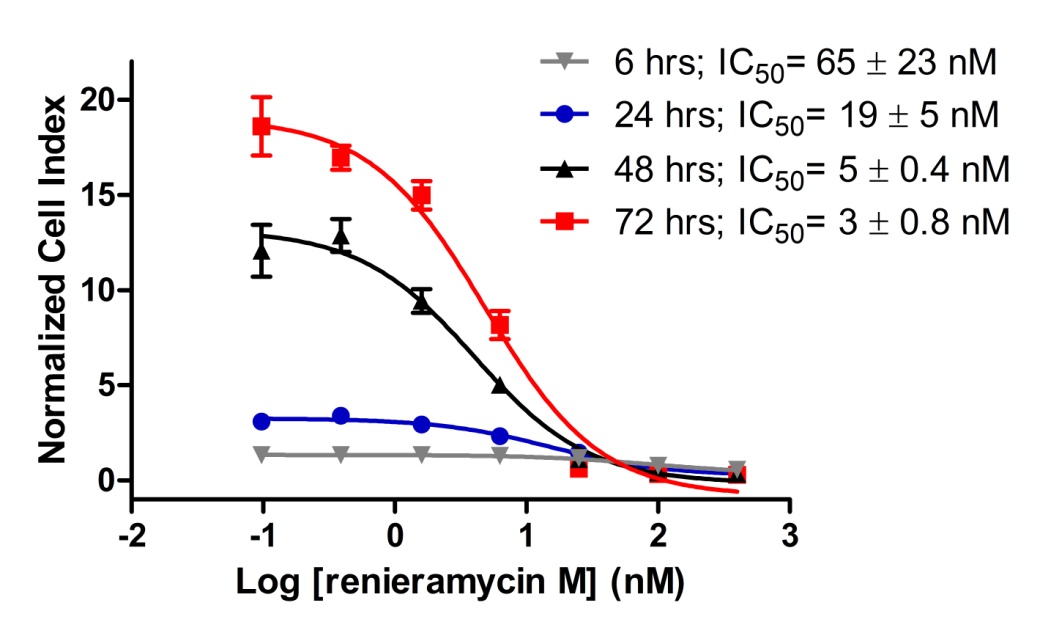
**

**A**

**
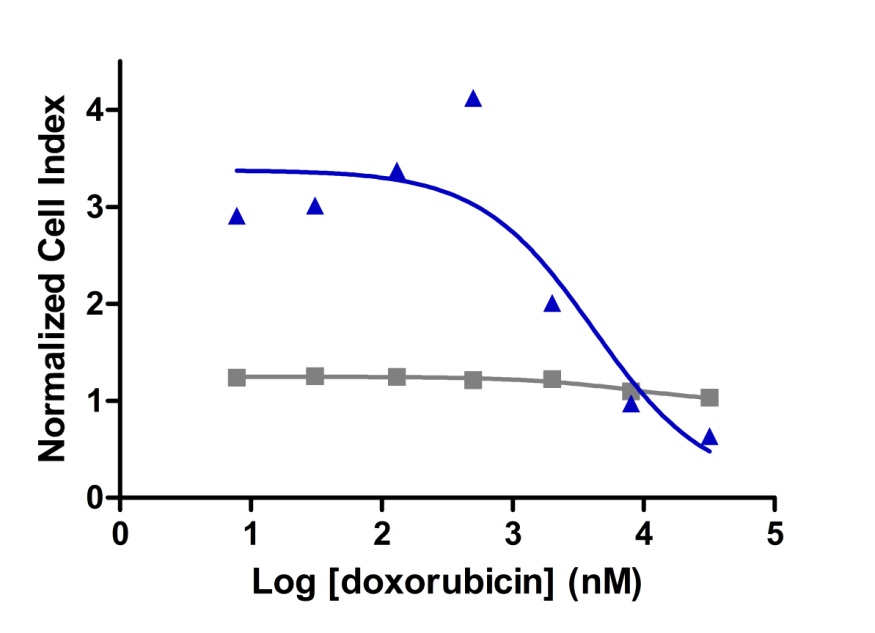
**

**B**

**
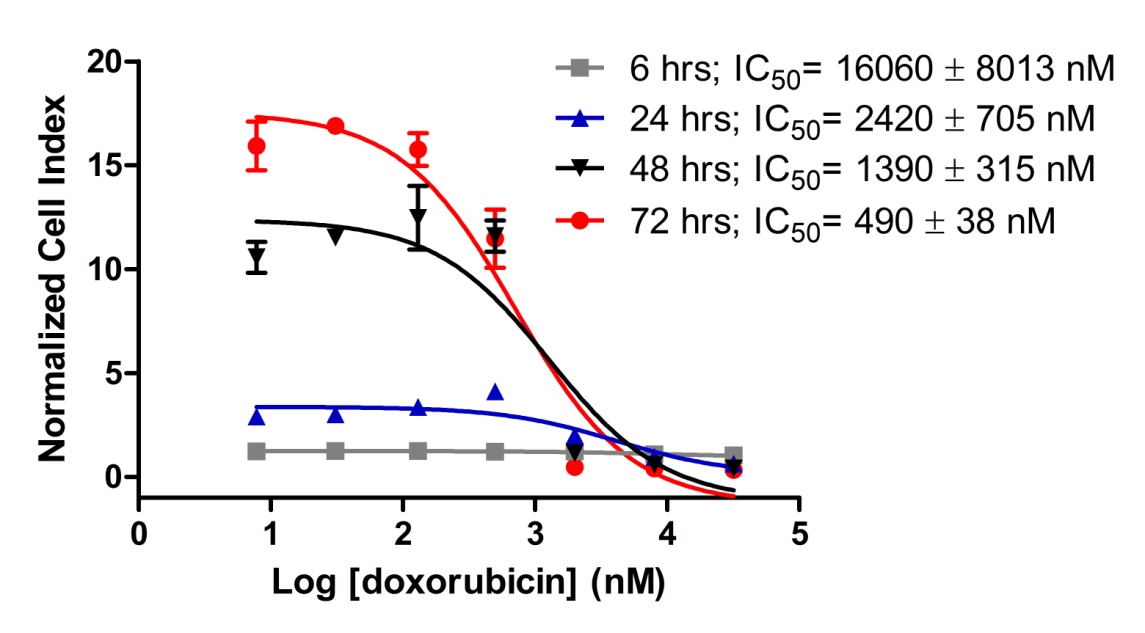
**

**Figure S3. Time-dependent cytotoxicity of RM and DOX using xCELLigence real-time cell analyzer (RTCA).** Concentration-response curves at 6, 24, 48, and 72 hours for RM- **(A)** and DOX-treated cells **(B)** were plotted, and the IC_50_ values were calculated using the sigmoidal dose-response variable slope equation of the RTCA software. Figures on the right are zoom versions of the concentration response curves at 6 and 24 hours. Data points are mean ± SD of (n = 4) of a representative trial. The IC_50_ values reported are mean ± SEM of five independent trials.


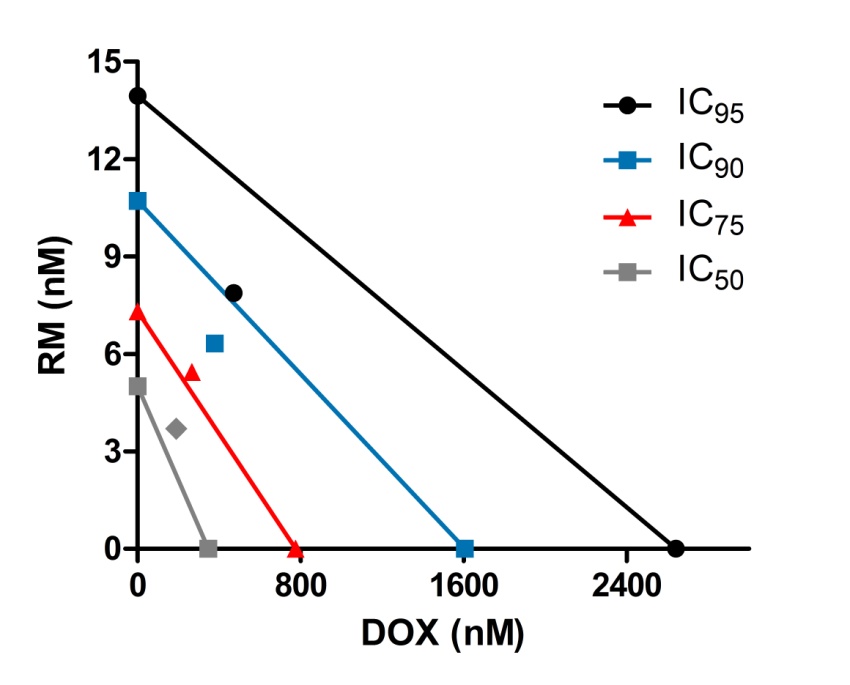

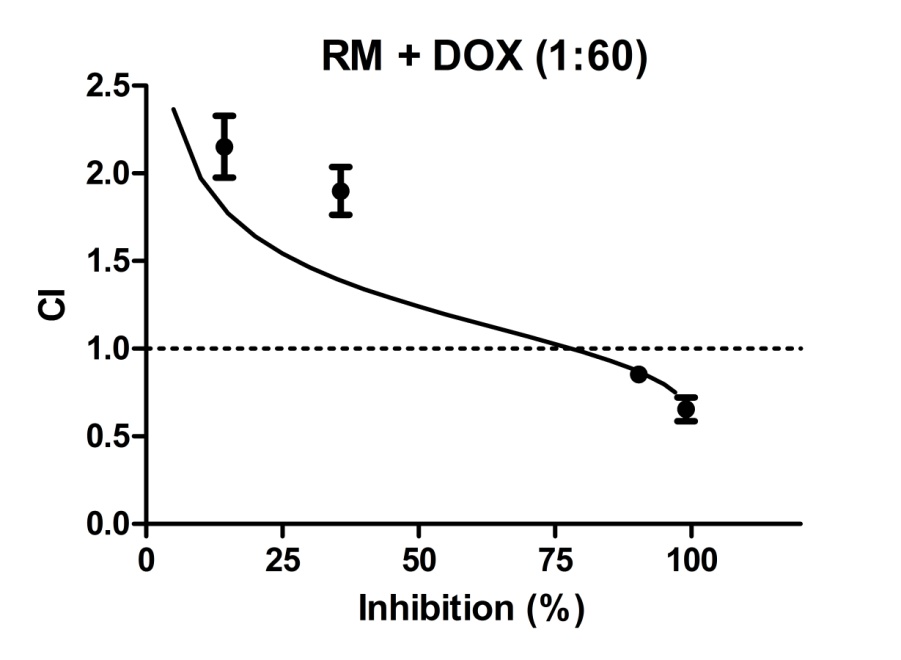

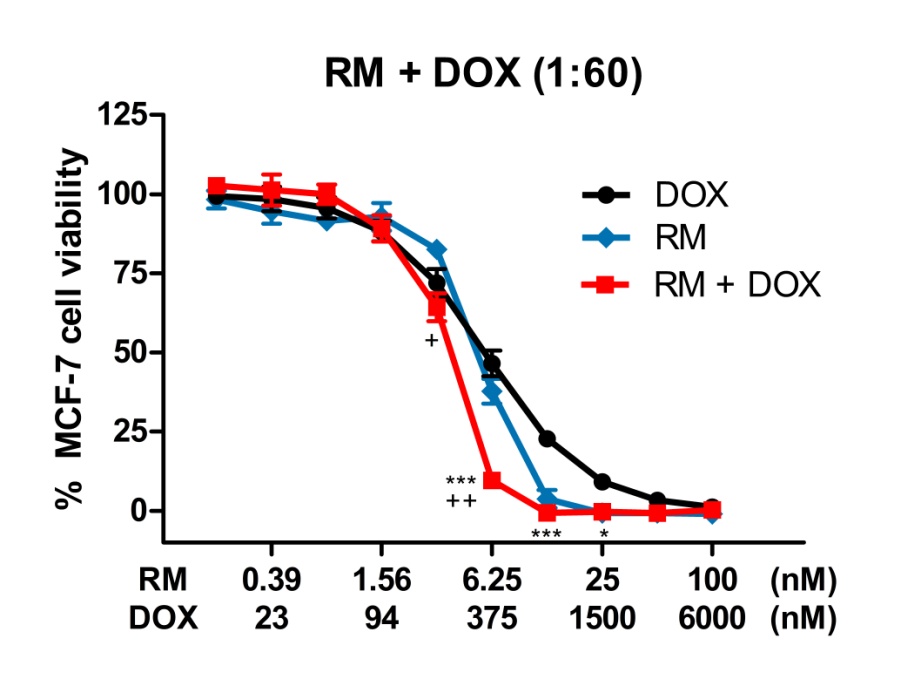


**Figure S4.** **Synergistic cytotoxicity of RM + DOX at equipotent molar ratio (1:60). (A)** Curve-shift analysis. MCF-7 cells were treated singly or concurrently with RM plus DOX at a constant molar ratio of 1:60 for 72 hours, and the cell viability was assessed using MTT cytotoxicity assay. Data points are mean ± SEM of three independent trials performed in quadruplicates. * *p* < 0.05, *** *p* < 0.0001 and + *p* < 0.05, ++ *p* < 0.001 compared to DOX alone and RM alone, respectively. **(B)** Combination Index (CI) plot. The CI values of experimental data points were calculated using CompuSyn software (Chou and Martin 2005), and the mean ± SEM of three independent trials were plotted as a function of the effect or percent Inhibition. CI < 1, = 1, > 1 mean synergism, additive, or antagonism, respectively. **(C)** Isobologram analysis. The diagonal lines denote additivity at different inhibition levels. The points are mean concentrations (n = 3) of RM and DOX required achieving 50%, 75%, 90%, and 95% inhibition.

**B**

**C**

**A**

**
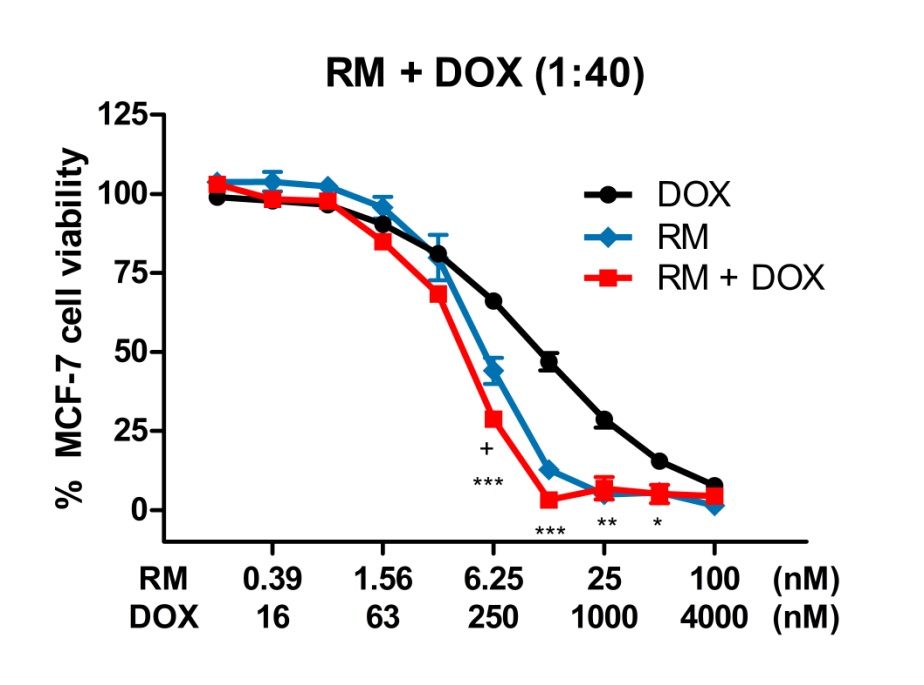

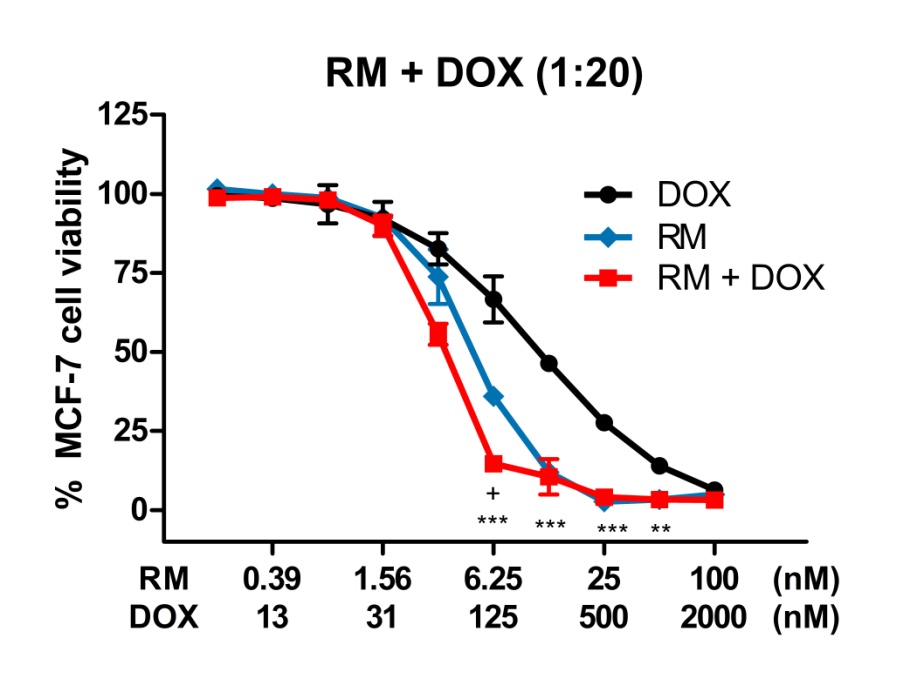

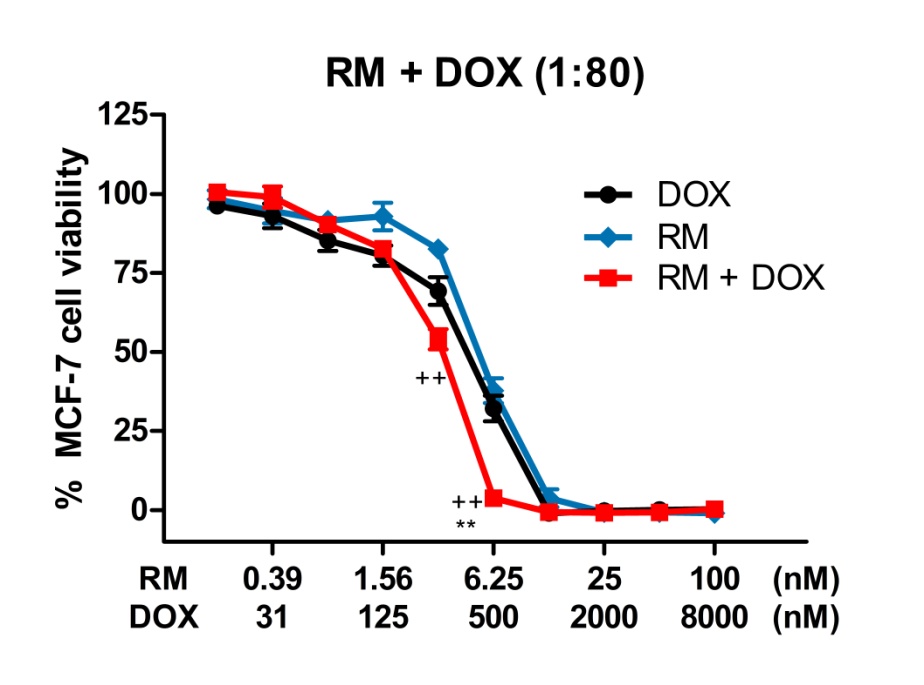

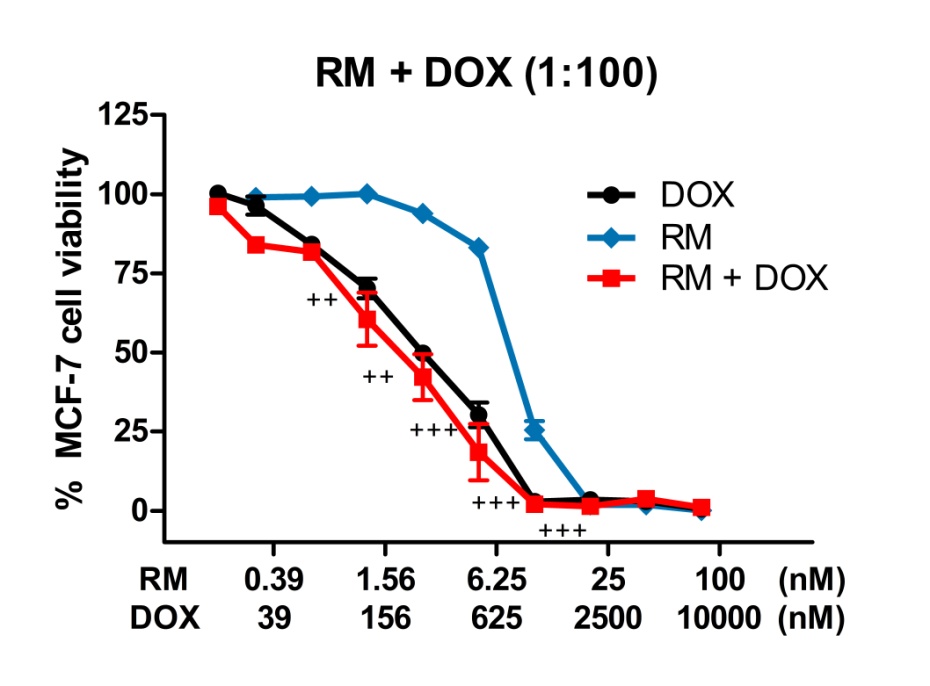
**

**Figure S5. Curve shift-analyses of the different combination ratios of RM + DOX.** Data points are mean ± SEM of three independent trials performed in quadruplicates * *p* < 0.05, ** *p* < 0.001, *** *p* < 0.0001 and + *p* < 0.05, ++ *p* < 0.001, +++ *p* < 0.0001 relative to DOX alone and RM alone, respectively (One-Way ANOVA/Bonferroni).

| **Drug Combination** | **Regimen** | **IC_50_ (nM)** | | | |  | | |  | **IC_95_ (nM)** | | | |  | | |
| --- | --- | --- | --- | --- | --- | --- | --- | --- | --- | --- | --- | --- | --- | --- | --- | --- |
|  |  | **Individual** | | **After**  **Combination** | | | **Fold-Change** | |  | **Individual** | | **After**  **Combination** | | | **Fold-Change** | |
|  |  | **RM** | **DOX** | **RM** | **DOX** | | **RM** | **DOX** |  | **RM** | **DOX** | **RM** | **DOX** | | **RM** | **DOX** |
| RM + DOX | 1:100 | 8 ± 0.3 | 323 ± 53 | 2 ± 0.4 | 221 ± 41 | | 4 | 1 |  | 32 ± 7 | 2490 ± 528 | 18 ± 3 | 2187 ± 113 | | 2 | 1 |
| RM + DOX | 1:80 | 5 ± 0.3 | 348 ± 41 | 3 ± 0.1 | 204 ± 10 | | 2 | 2 |  | 14 ± 1 | 2291 ± 366 | 6 ± 1 | 514 ± 45 | | 2 | 4 |
| RM + DOX | 1:60 | 5 ± 0.3 | 348 ± 41 | 3 ± 0.1 | 198 ± 9 | | 2 | 2 |  | 14 ± 1 | 2291 ± 366 | 8 ± 1 | 479 ± 45 | | 2 | 5 |
| RM + DOX | 1:50 | 7 ± 0.4 | 359 ± 58 | 3 ± 0.3 | 137 ± 17 | | 3 | 3 |  | 27 ± 5 | 5868 ± 1895 | 11 ± 1 | 604 ± 74 | | 3 | 10 |
| RM + DOX | 1:40 | 7 ± 0.7 | 451 ± 26 | 4 ± 0.3 | 142 ± 13 | | 2 | 3 |  | 31 ± 9 | 8557 ± 1705 | 13 ± 1 | 576 ± 21 | | 3 | 15 |
| RM + DOX | 1:20 | 4 ± 0.1 | 190 ± 31 | 3 ± 0.1 | 64 ± 6 | | 1 | 3 |  | 25 ± 9 | 2794 ± 779 | 14 ± 4 | 283 ± 86 | | 2 | 10 |

**Table S1.** **Comparison of IC_50_ and IC_95_ of single and combination drug treatments.**

Values are mean ± SEM of multiple independent trials (n ≥ 3). Fold-change was calculated by dividing IC_95_ of the individual drugs to the resulting IC_95_ after combination.


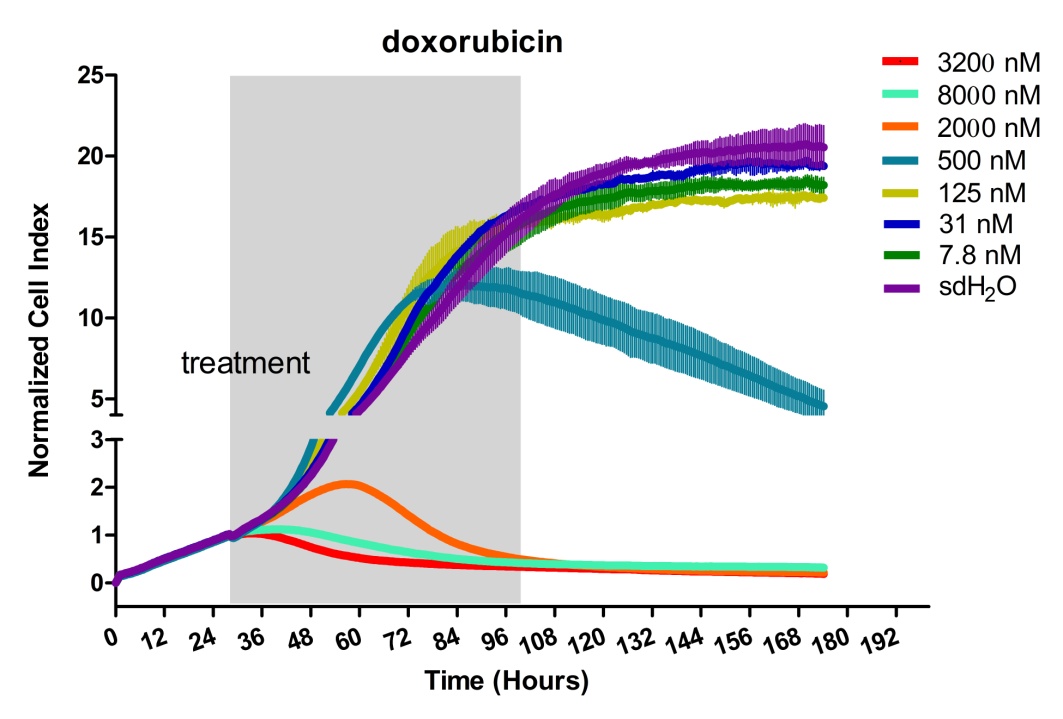

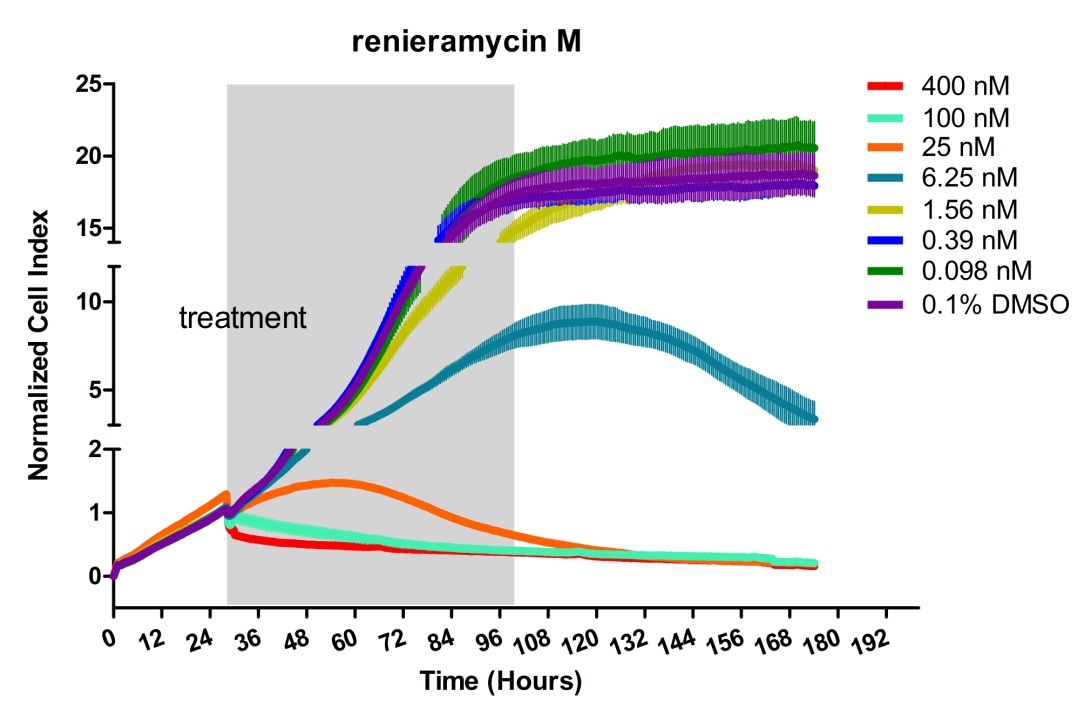


**A**

**B**


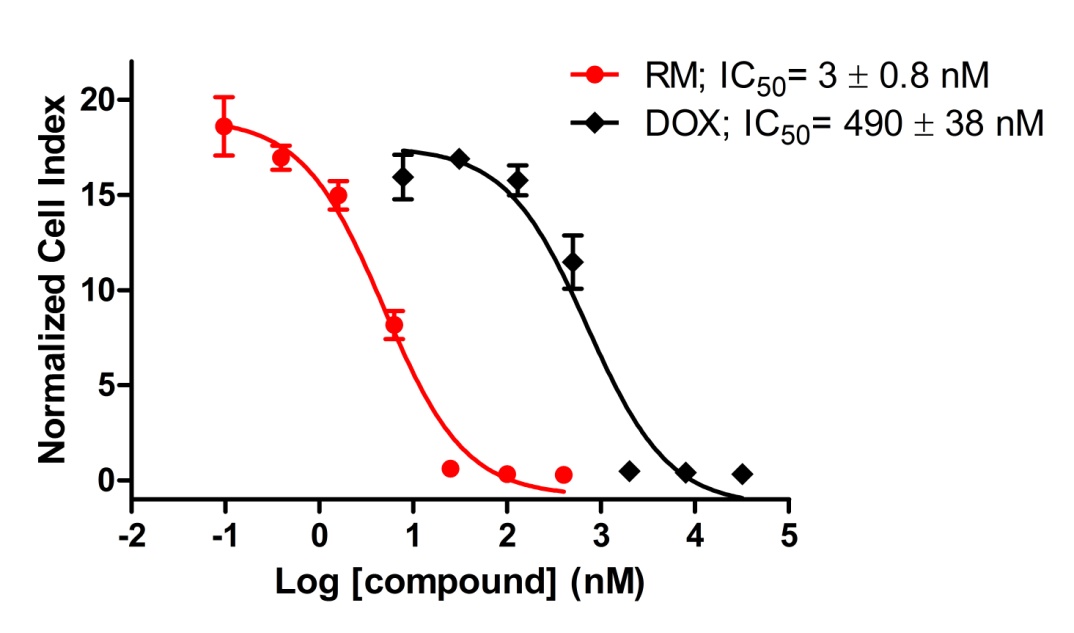


**C**


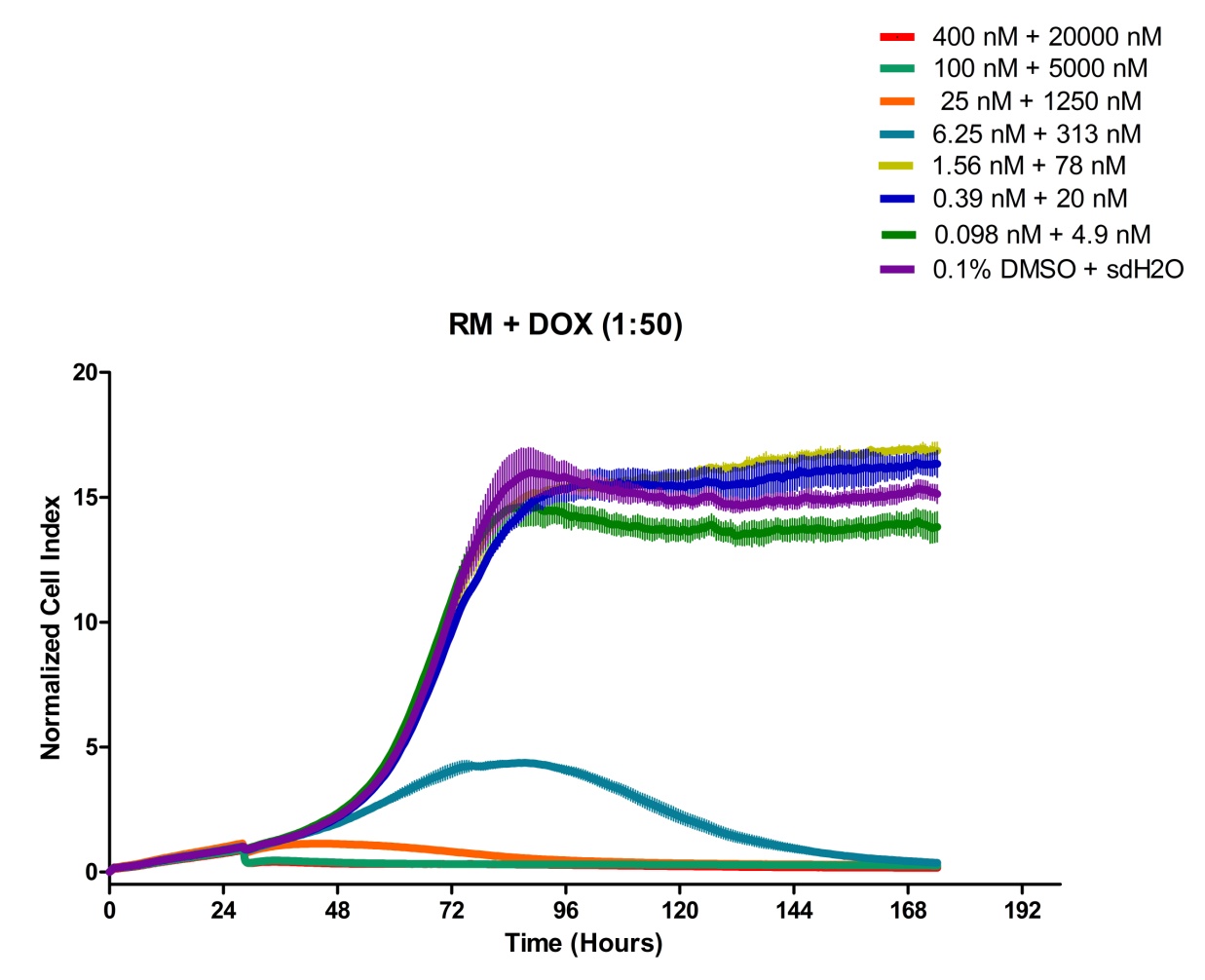


treatment


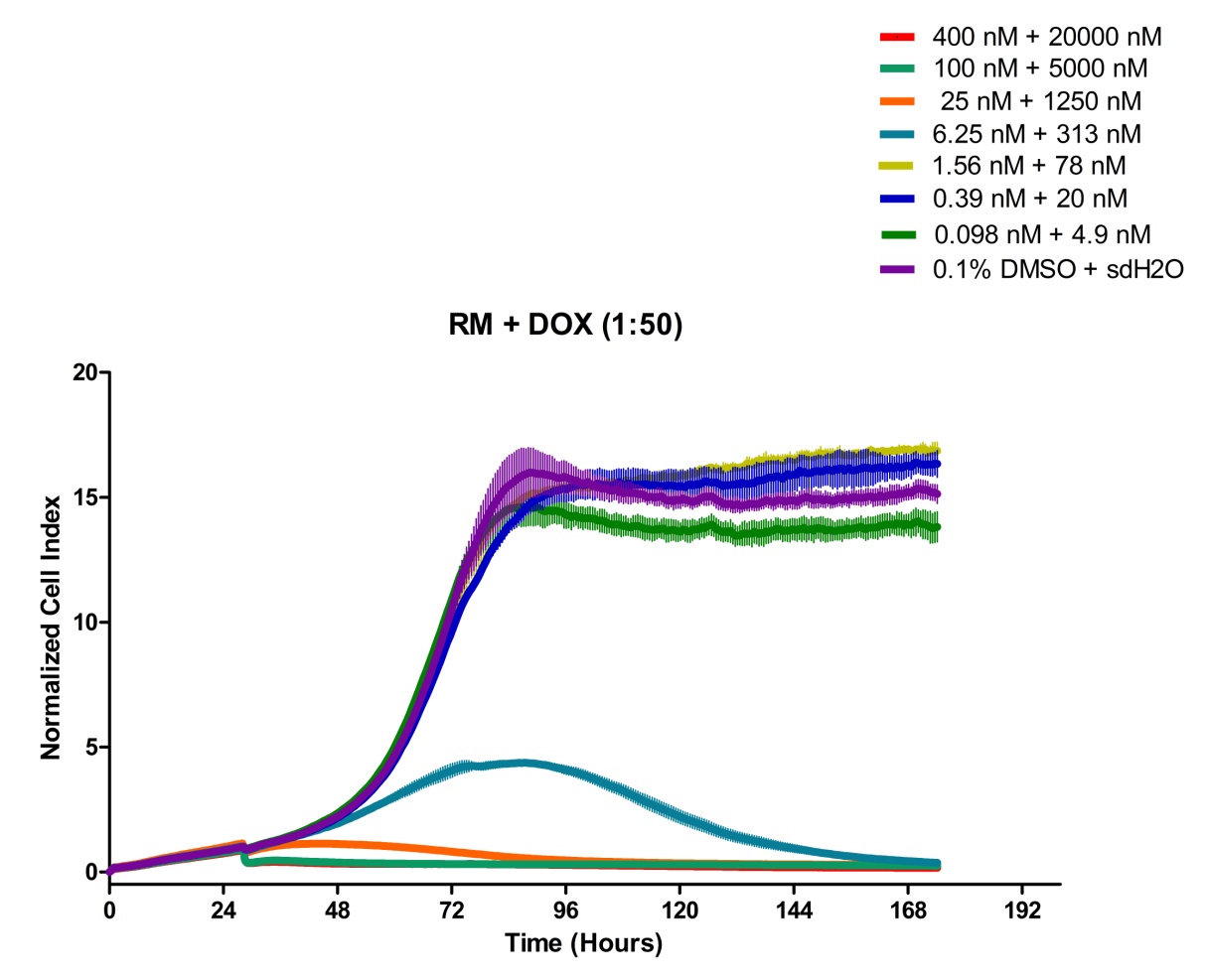


**D**

**Figure S6.** **Dynamic monitoring of the effects of RM and DOX, singly on MCF-7 breast cancer cells.** Kinetic cell response profiles of MCF-7 cells treated singly with **(A)** DOX or **(B)** RM. MCF-7 cells were treated with the indicated concentrations of RM, DOX, and vehicle controls (0.1% DMSO and sdH_2_O) and monitored continuously for 7 days using the xCELLigence System (left panel). The Cell Indices were normalized at the time of compound addition indicated by the arrows. The gray area denotes the 72-hour exposure period. Data points are mean ± SD of (n = 4) of a representative trial. (**C)** Calculation of the IC_50_ in real-time after 72 hours of treatment. Data points are mean ± SD of (n = 4) of a representative trial. The IC_50_ values reported are mean ± SEM of five independent trials. **(D)** Full kinetic cell response profile of MCF-7 cells treated with RM+ DOX (1:50) for 7 days.

**Figure S7.** **Morphological changes in MCF-7 breast cancer cells treated singly, and in combination of RM and DOX for 30 and 48 hrs. (A)** RM-treated cells. **(B)** DOX-treated cells. **(C)** RM + DOX (1:50). Scale bar = 20 μm.

**
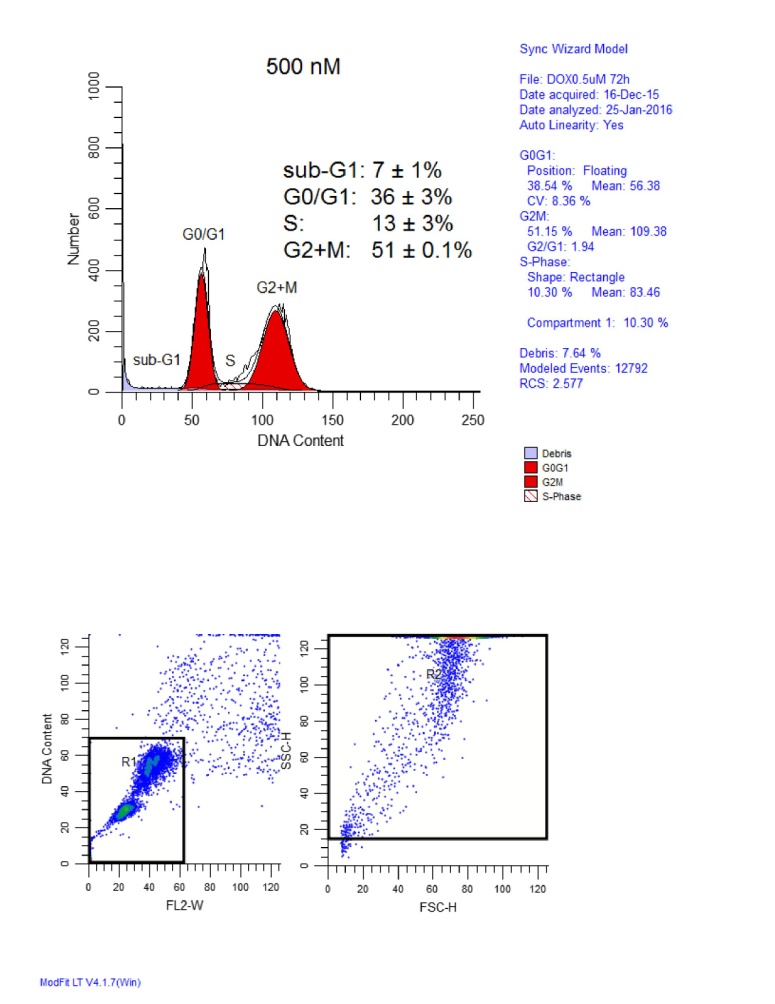

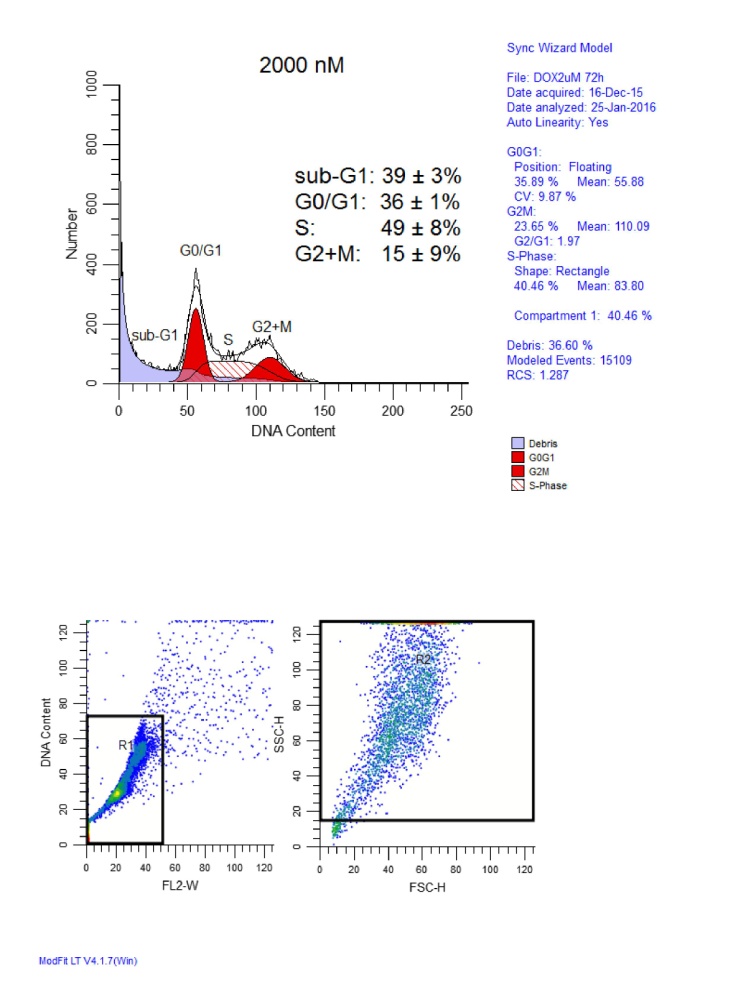

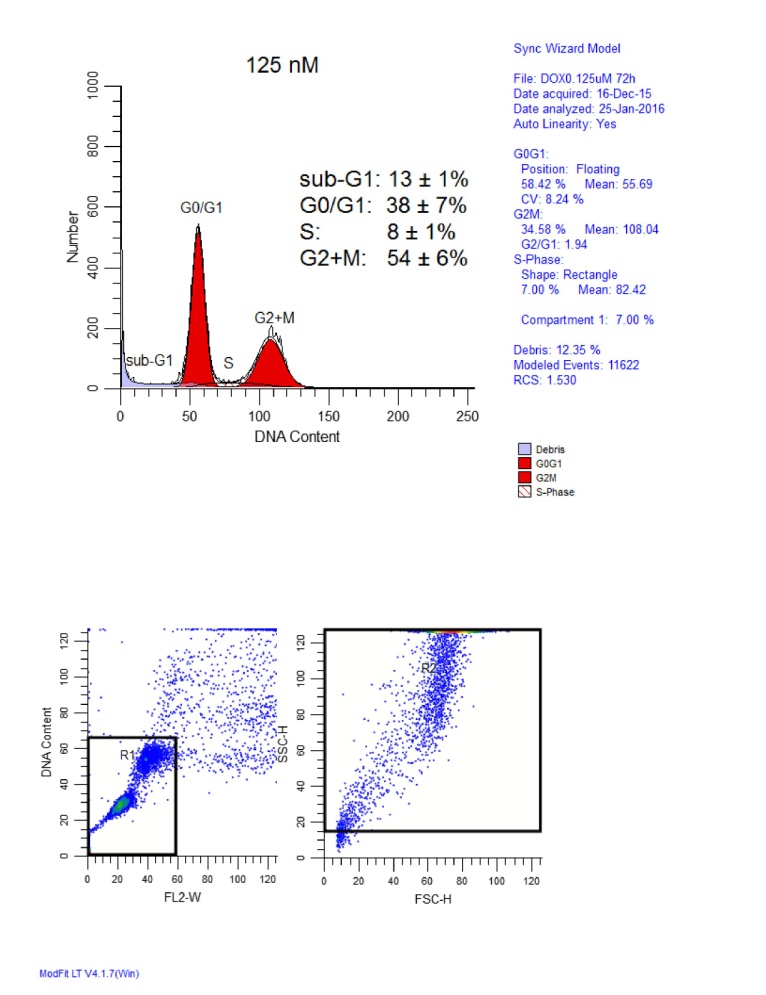

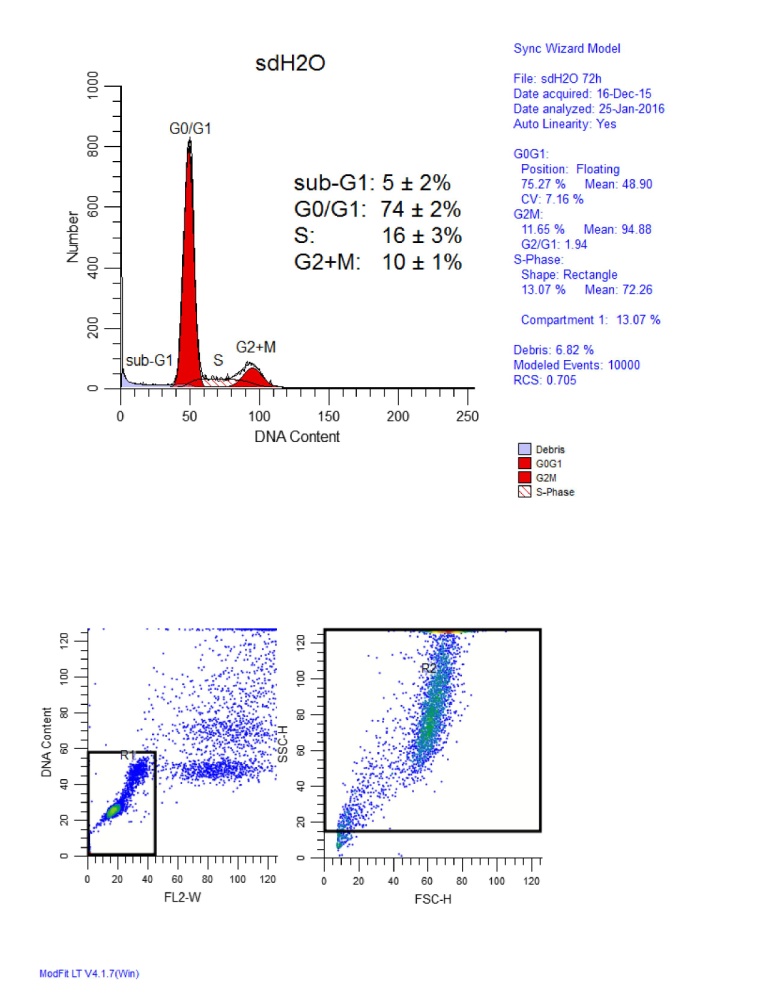

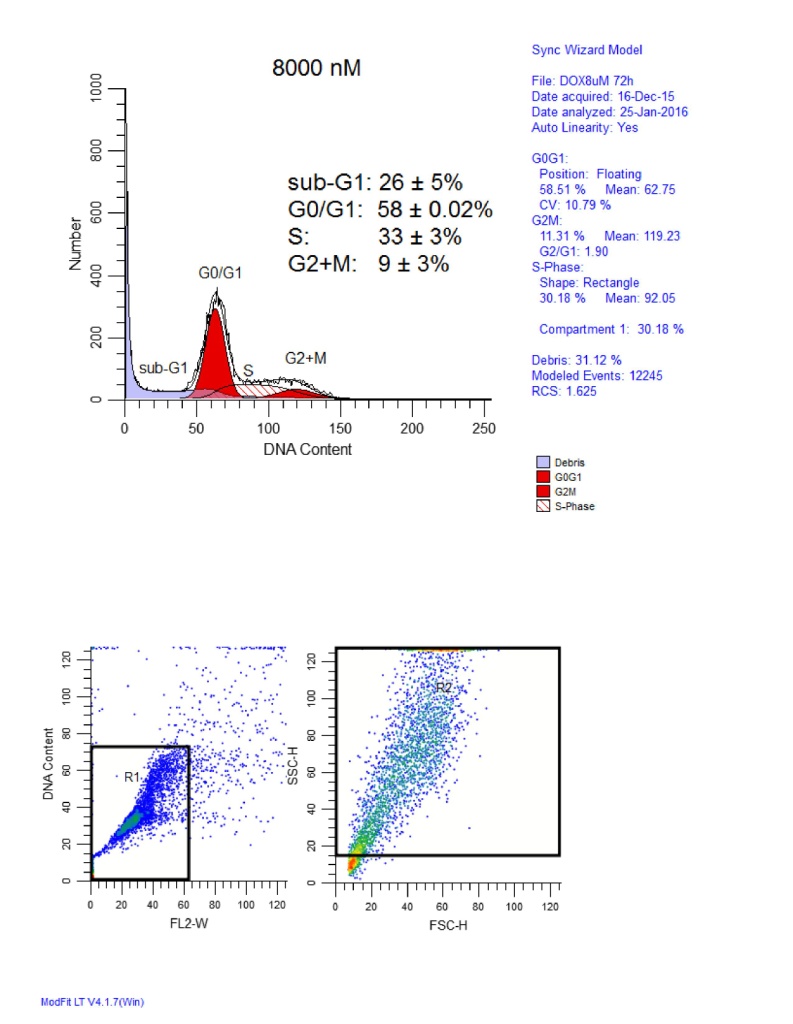

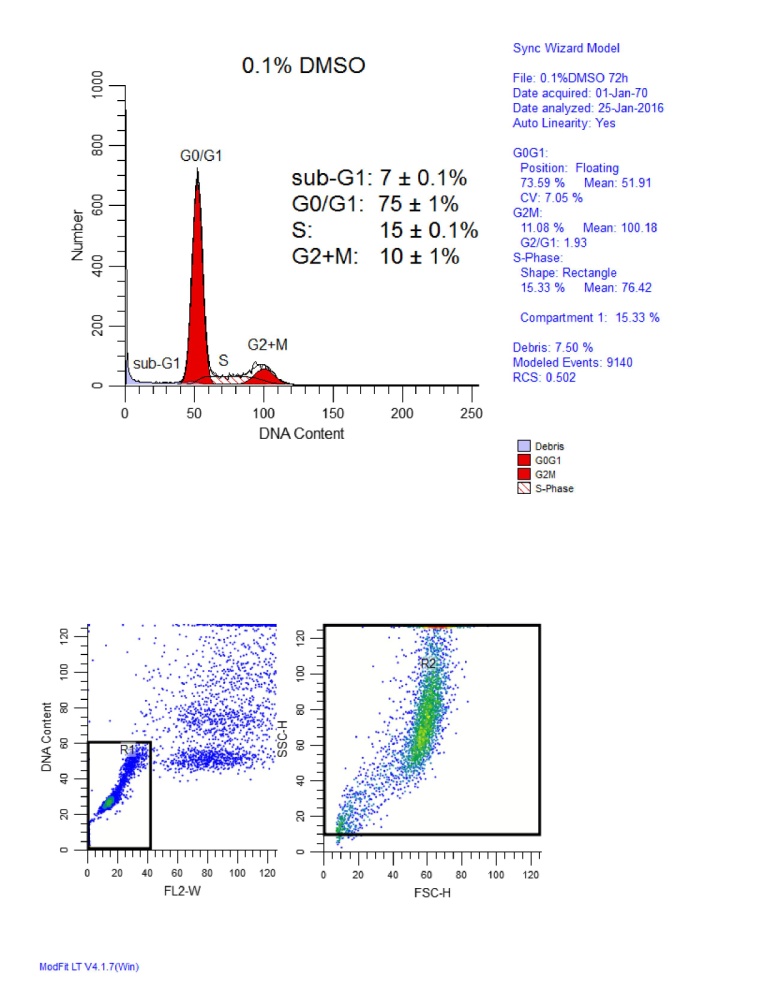

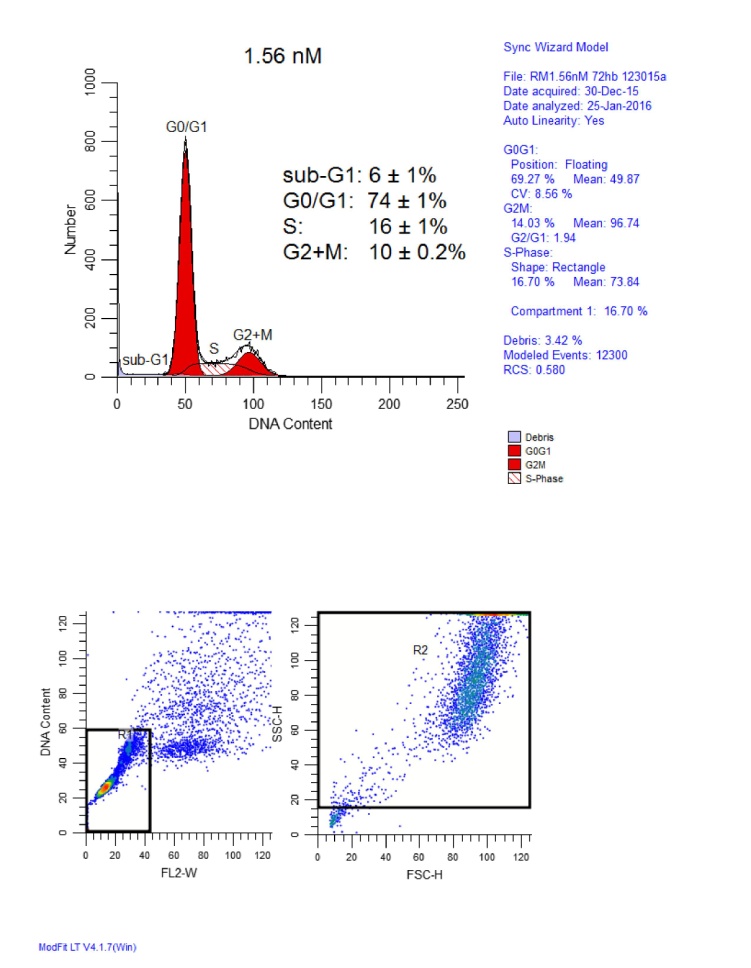

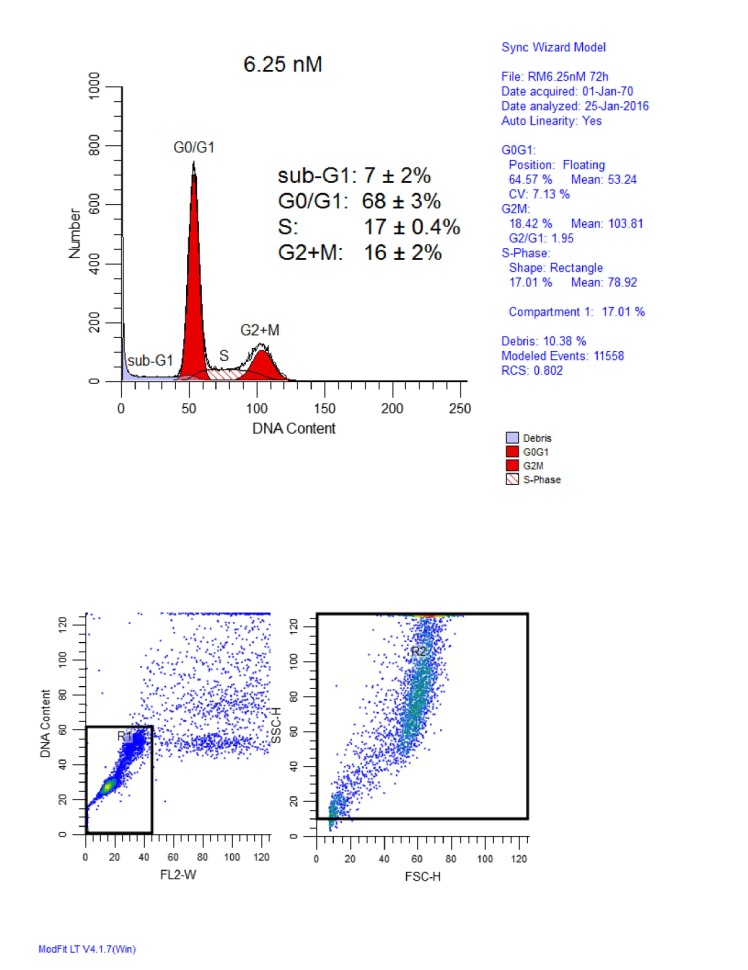

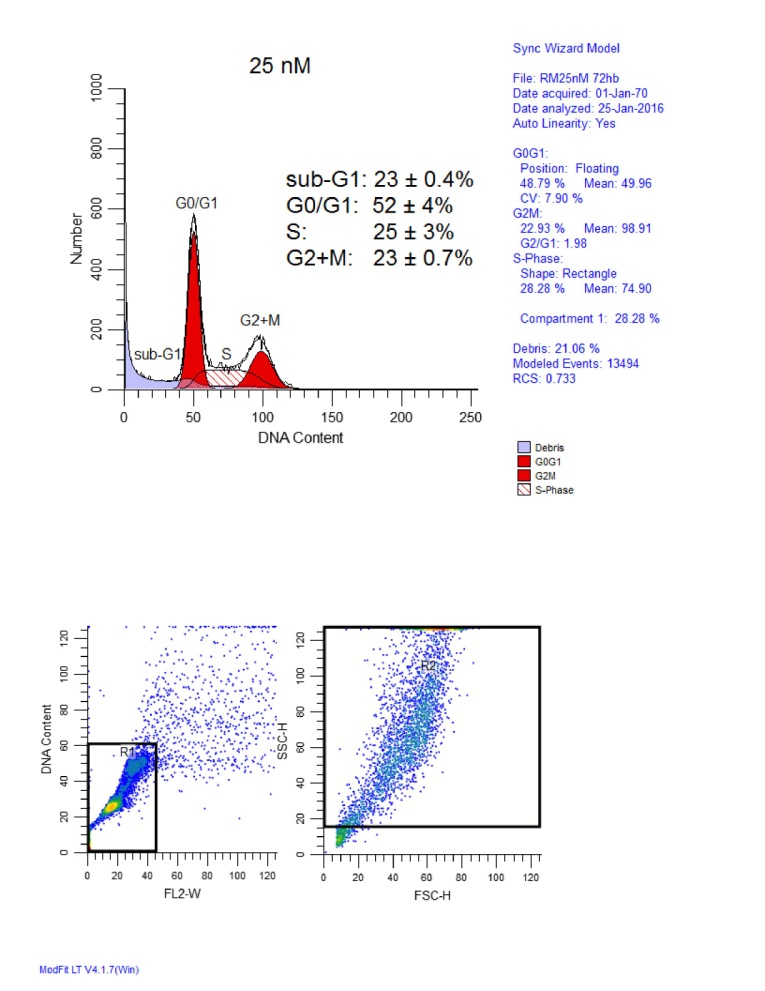

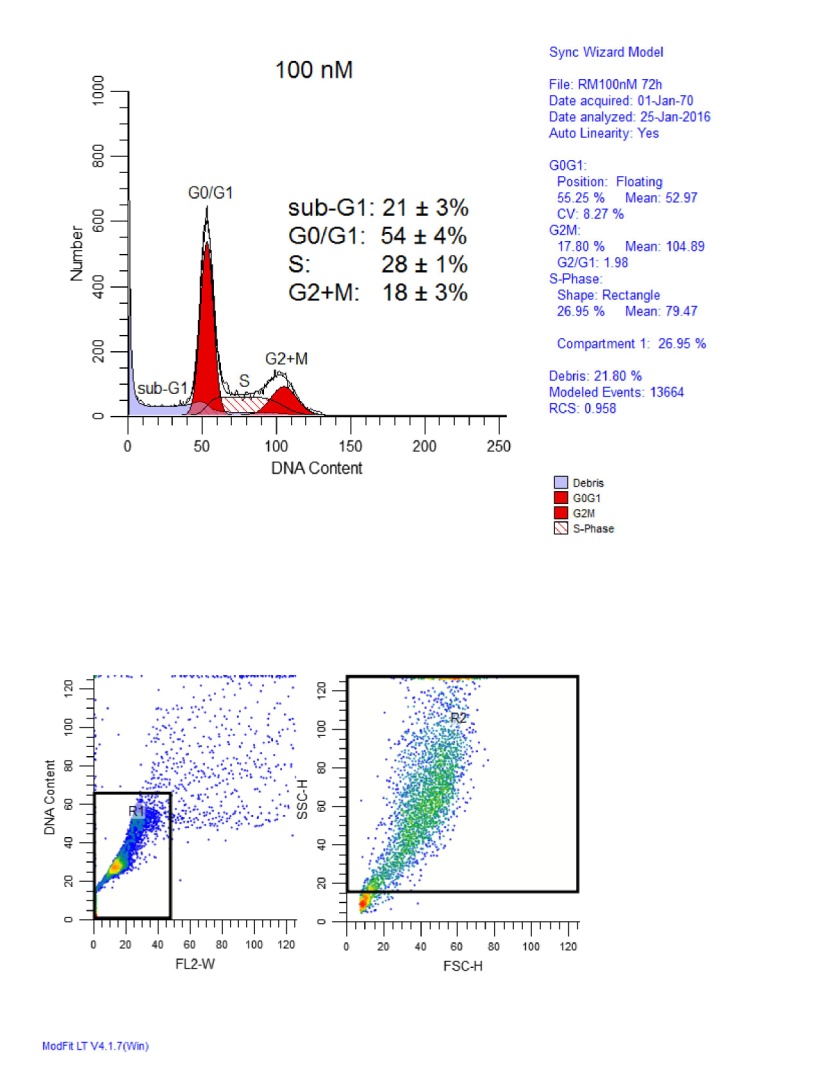
**

*******

*******

*******

*******

*******

*******

*******

******

*****

*****

******

******

******

******

******

*****

******

*****

**B.**

**DOX concentration**

**RM concentration**

**A.**

**
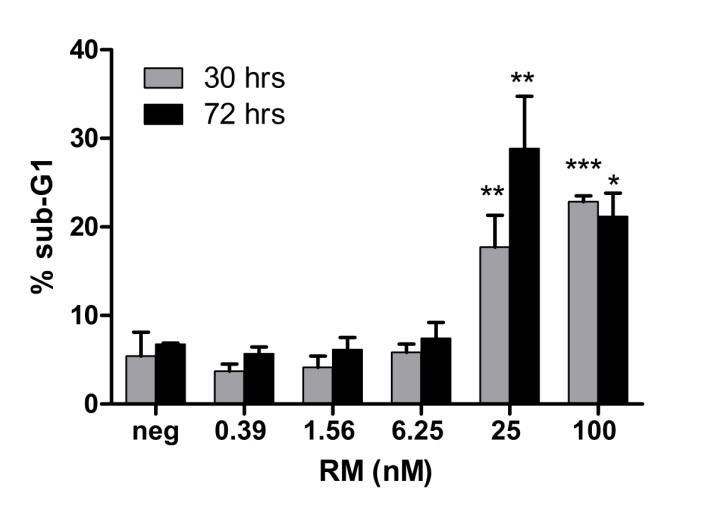
**

**C.**

**
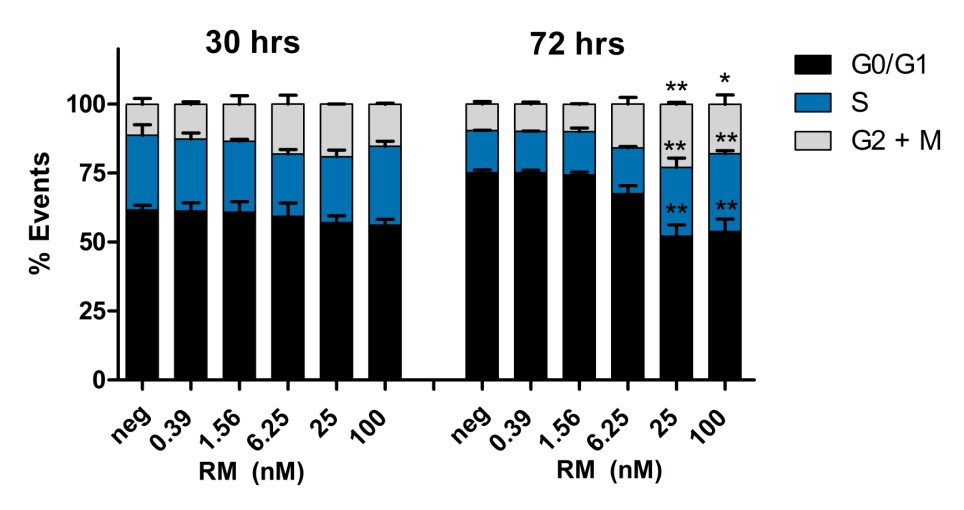
**

**
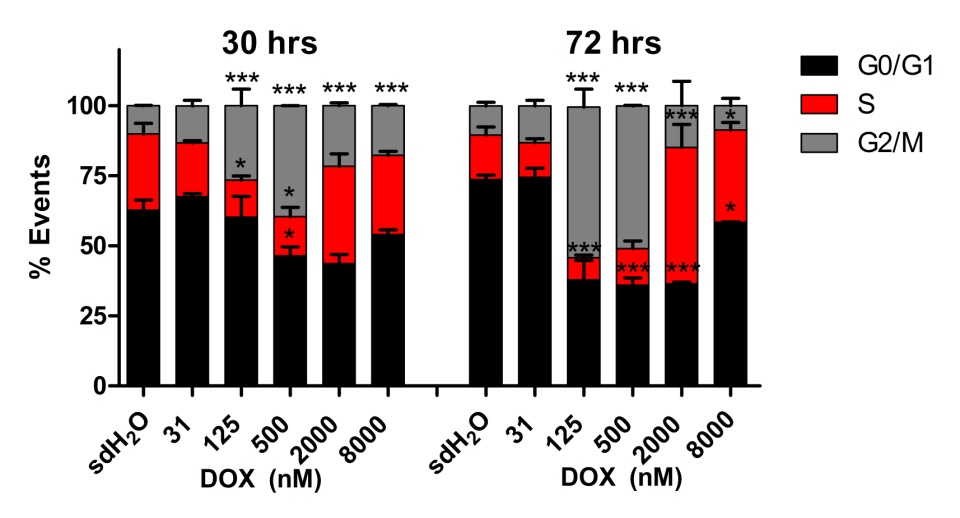

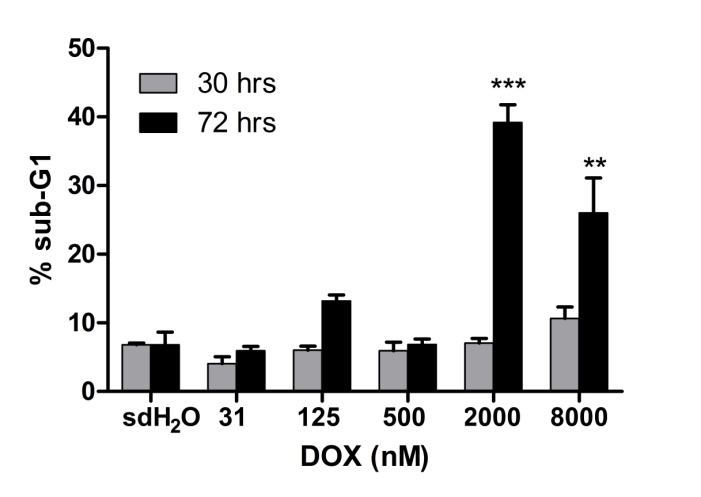
**

**D.**

**Figure S8.** **Effects of RM alone and DOX alone on MCF-7 cell cycle.** **(A-B)** DNA content analysis using propidium iodide (PI) 72 h-post treatment. RM induced an S and G2 arrest at high concentrations (25-100 nM). Lower concentrations of DOX (125 to 500 nM) induced a G2 arrest, while higher concentrations caused a G1 and S arrest. The histograms are representative of three independent trials. The percentages reported are mean + SEM (n = 3). **(C-D)** Time-dependent effects of RM and DOX on the cell cycle stages. 0.1% DMSO (neg) and sdH_2_O served as negative controls. Bars are mean + SEM (n = 3). * *p* < 0.05, ** *p* < 0.001, *** *p* < 0.0001 (One Way ANOVA/Dunnett’s Multiple Comparison Test).


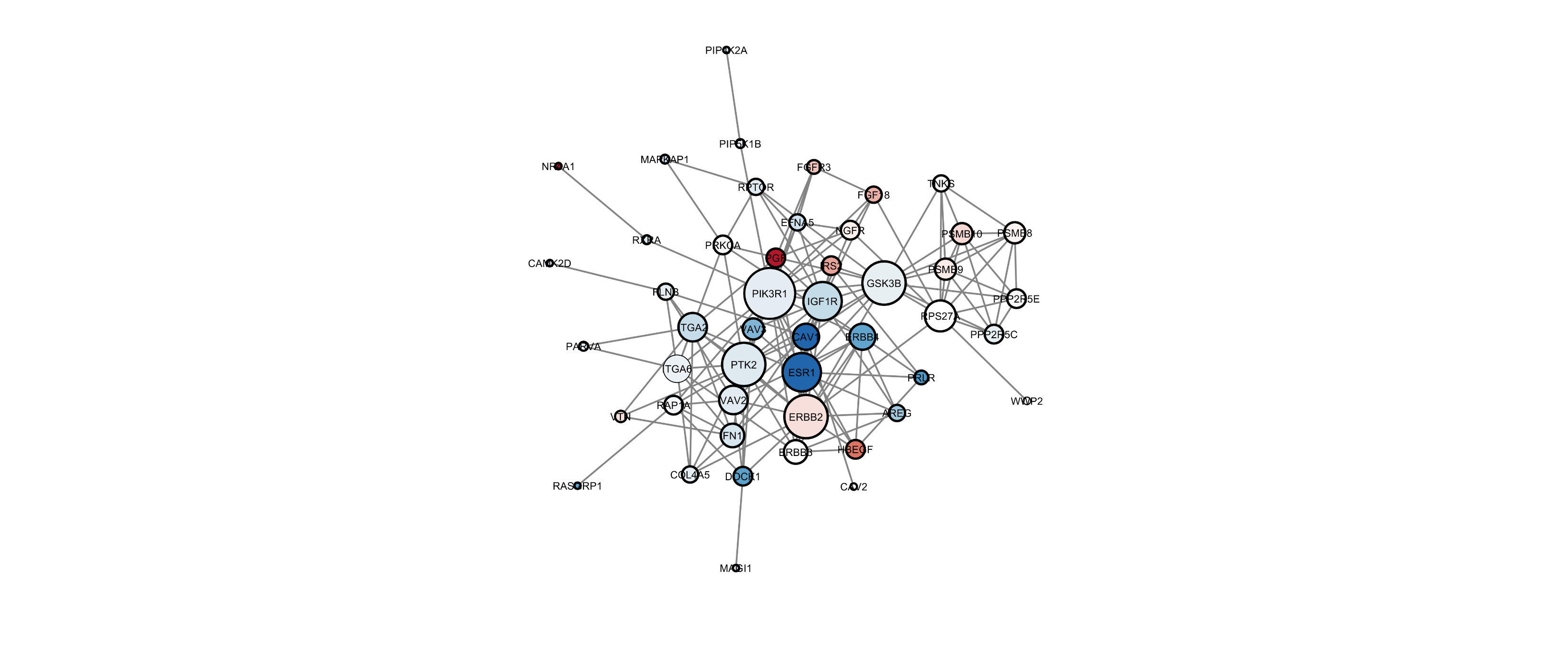

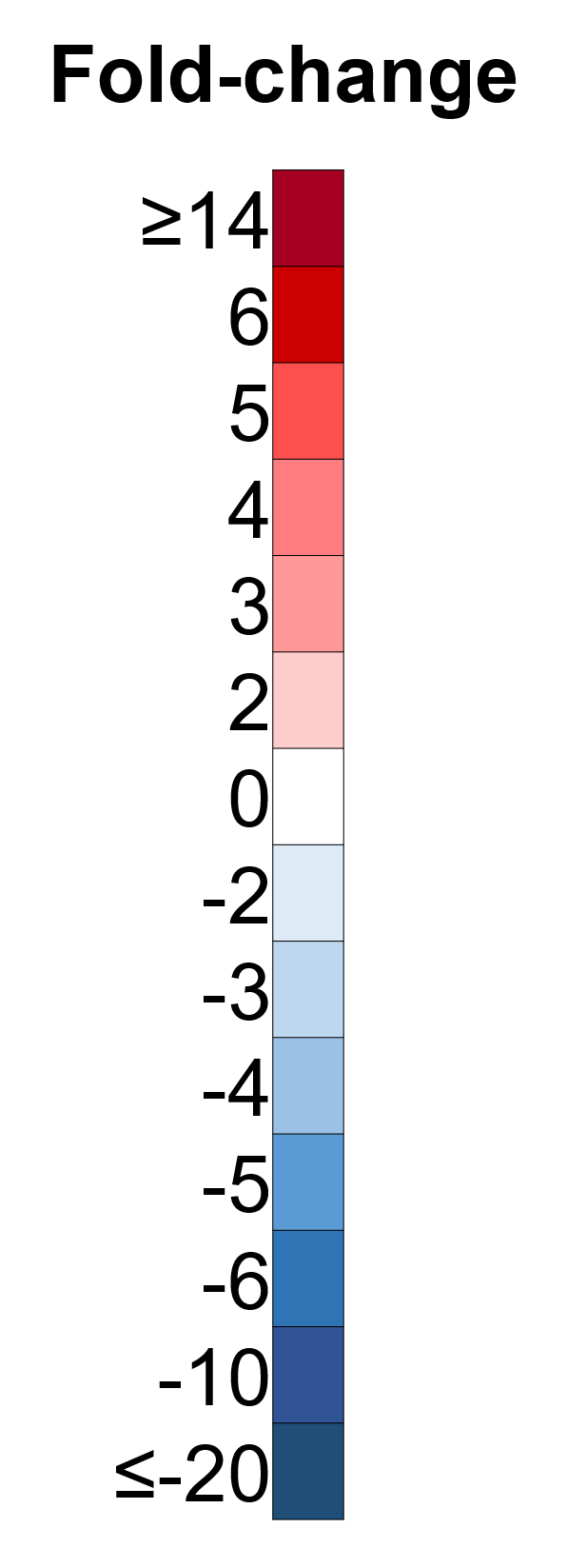

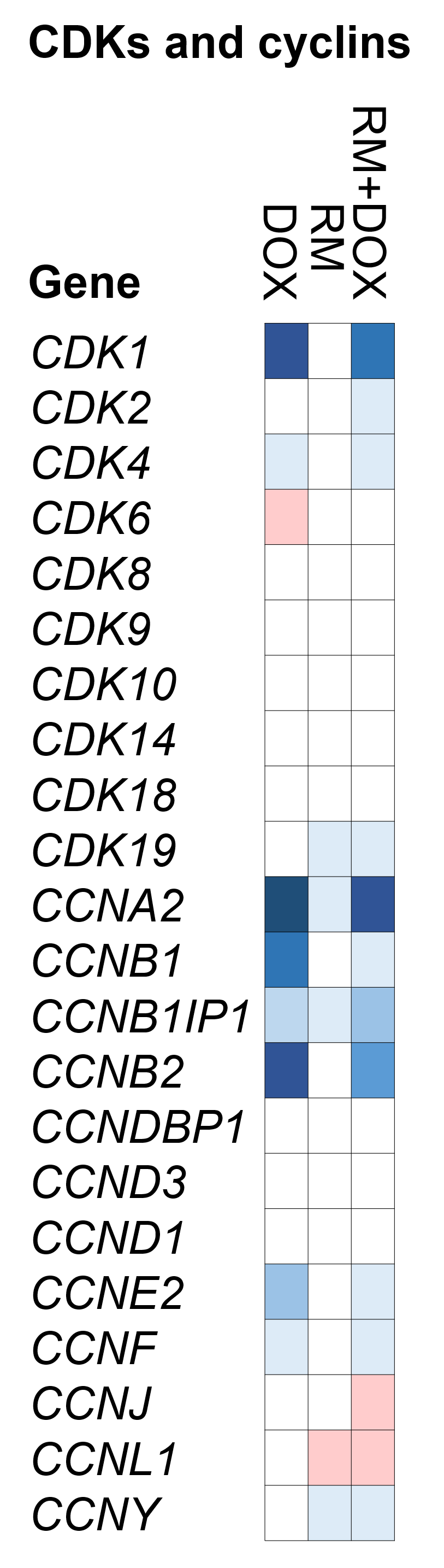


**B.**

**A.**

**Figure S9. (A)** Cyclin-dependent kinases (CDKs) and cyclins affected in MCF-7 after single and combination treatment with RM (6.25 nM) and DOX (3.13 nM). **(B)** Molecular network of genes involved in ErbB/PI3K-Akt and integrin/focal adhesion pathways using STRING database and Cytoscape v.3.6.1. Each node represents a single gene. Size of the nodes, border width, and intensity of color correspond to degree of connectivity, FDR *p*-value <0.05 and fold-change, respectively. Blue = downregulation; red = upregulation.
